# ALKBH5-mediated m^6^A demethylation of *Runx2* mRNA promotes extracellular matrix degradation and intervertebral disc degeneration

**DOI:** 10.1186/s13578-024-01264-y

**Published:** 2024-06-14

**Authors:** Yu Lei, Enyu Zhan, Chao Chen, Yaoquan Hu, Zhengpin Lv, Qicong He, Xuenan Wang, Xingguo Li, Fan Zhang

**Affiliations:** Department of Orthopedics, The First Affiliated Hospital of Kunming Medical University, 295 Xichang Rd, Wuhua District, Kunming, Yunnan 650032 China

**Keywords:** m^6^A demethylation, ALKBH5, YTHDF1, Runx2, MMP, ADAMTS, Intervertebral disc degeneration

## Abstract

**Background:**

N6-methyladenosine (m^6^A) methylation is a prevalent RNA modification implicated in various diseases. However, its role in intervertebral disc degeneration (IDD), a common cause of low back pain, remains unclear.

**Results:**

In this investigation, we explored the involvement of m^6^A demethylation in the pathogenesis of IDD. Our findings revealed that ALKBH5 (alkylated DNA repair protein AlkB homolog 5), an m^6^A demethylase, exhibited upregulation in degenerative discs upon mild inflammatory stimulation. ALKBH5 facilitated m^6^A demethylation within the three prime untranslated region (3′-UTR) of *Runx2* mRNA, consequently enhancing its mRNA stability in a YTHDF1 (YTH N6-methyladenosine RNA binding protein F1)-dependent manner. The subsequent elevation in Runx2 expression instigated the upregulation of ADAMTSs and MMPs, pivotal proteases implicated in extracellular matrix (ECM) degradation and IDD progression. In murine models, subcutaneous administration of recombinant Runx2 protein proximal to the lumbar disc in mice elicited complete degradation of intervertebral discs (IVDs). Injection of recombinant MMP1a and ADAMTS10 proteins individually induced mild to moderate degeneration of the IVDs, while co-administration of MMP1a and ADAMTS10 resulted in moderate to severe degeneration. Notably, concurrent injection of the Runx2 inhibitor CADD522 with recombinant Runx2 protein did not result in IVD degeneration in mice. Furthermore, genetic knockout of ALKBH5 and overexpression of YTHDF1 in mice, along with lipopolysaccharide (LPS) treatment to induce inflammation, did not alter the expression of Runx2, MMPs, and ADAMTSs, and no degeneration of the IVDs was observed.

**Conclusion:**

Our study elucidates the role of ALKBH5-mediated m^6^A demethylation of *Runx2* mRNA in activating MMPs and ADAMTSs, thereby facilitating ECM degradation and promoting the occurrence of IDD. Our findings suggest that targeting the ALKBH5/Runx2/MMPs/ADAMTSs axis may represent a promising therapeutic strategy for preventing IDD.

**Supplementary Information:**

The online version contains supplementary material available at 10.1186/s13578-024-01264-y.

## Background

Intervertebral disc (IVD) degeneration (IDD) represents the predominant cause of low back pain (LBP) associated with aging [1, 2]. This progressive process involves the gradual loss of extracellular matrix (ECM) constituents, including collagen and proteoglycans, coupled with alterations in the morphology and functionality of disc cells [3]. Multiple mechanisms have been proposed to contribute to IDD, encompassing mechanical overload, genetic predisposition, inflammation, and oxidative stress [1, 2, 4, 5]. Mechanical overload induces stress and microtrauma on the discs, resulting in the release of pro-inflammatory cytokines such as tumor necrosis factor-alpha (TNF-α), interleukin-1 beta (IL-1β), and IL-6, along with chemokines, and activation of matrix-degrading enzymes including a disintegrin-like and metalloprotease with thrombospondin motifs (ADAMTSs) and matrix metallopeptidases (MMPs) [6–8]. Genetic factors also play a role in IDD by modulating the expression of genes implicated in disc metabolism and maintenance [9]. Concurrently, inflammation and oxidative stress further exacerbate disc degeneration by fostering the generation of reactive oxygen species (ROS), prompting cell death and tissue damage [10, 11]. These pathological processes culminate in diminished disc height, herniation, and nerve compression, manifesting as LBP and associated symptoms [1, 2, 4, 5]. Moreover, managing IDD remains challenging, as curative interventions are lacking, and current treatments primarily aim to alleviate symptoms and enhance quality of life [12–14]. Conservative therapies, including physical therapy, pain management, and exercise, can alleviate symptoms, while surgical intervention may be necessary in severe cases [12–14]. Elucidating the mechanisms underpinning IDD holds promise for shedding light on its pathogenesis and fostering the development of innovative therapeutic approaches.

Runt-related transcription factor 2 (Runx2) stands as a pivotal regulator of bone development and homeostasis, and emerging evidence has implicated its role in the pathogenesis of IDD [15–17]. In response to glucose stimulation, Runx2 orchestrates the recruitment of both PPARgamma coactivator 1alpha (PGC-1α) and CREB-binding protein (CBP), facilitating the transactivation of ADAMTS4/5 expression [18]. Furthermore, in aged mice, Runx2 engages a histone acetyltransferase p300 and the coactivator NCOA1 (nuclear receptor coactivator 1) to form a complex, thereby transactivating the expression of 14 *MMPs* (*MMP1b, -2, -3, -7, -8, -9, -10, -12, -13, -15, -16, -23, -27*, and − *28*) [19]. Elevated levels of Runx2 have been observed in degenerated IVDs, where it promotes the expression of matrix-degrading enzymes, including MMPs and ADAMTSs, within disc cells [18–20]. Additionally, Runx2 can activate the Wnt/β-catenin signaling pathway, which has been implicated in IVD degeneration [21]. Despite the observed upregulation of Runx2 in degenerative discs, the precise mechanism underlying its overexpression remains elusive.

Methylation of adenosine at the N6 position (m^6^A) represents one of the most common RNA modifications and has emerged as a pivotal regulator in numerous biological processes, encompassing RNA stability, splicing, translation, and degradation [22, 23]. This modification undergoes dynamic and reversible regulation orchestrated by a complex interplay among writers, erasers, and readers. Methyltransferase-like 3 (METTL3), in concert with its cofactors METTL14 and Wilms’ tumor 1-associated protein (WTAP), is responsible for m^6^A deposition in mRNA [22, 23]. Conversely, demethylation of m^6^A is governed by mass and obesity-associated protein (FTO) and α-ketoglutarate-dependent dioxygenase alkB homolog 5 (ALKBH5) [22, 23]. The functional impact of m^6^A modification on RNA metabolism is predominantly mediated by a diverse array of m^6^A-binding proteins, commonly referred to as “readers” [22, 23]. These readers encompass a spectrum of proteins, including members of the YT521-B homology (YTH) domain family (YTHDC1-2 and YTHDF1-3), heterogeneous nuclear ribonucleoproteins (HNRNPs), and insulin-like growth factor 2 mRNA-binding proteins (IGF2BPs) [22, 23]. Typically, m^6^A modification occurs at specific RNA sequences denoted as RRACH (where R denotes A, G, or U; and H denotes A, C, or U), predominantly enriched in the 3′-untranslated regions (3′-UTRs) [22, 23]. Dysregulation of m^6^A methylation has been implicated in a spectrum of human diseases, spanning cancer [23], obesity [24], neurological disorders [25], and IDD [26]. Hence, deciphering the ramifications of m^6^A methylation holds promise for shedding light on the pathogenesis and therapeutic avenues for these disorders.

In this study, we assessed the expression levels of RNA m^6^A methyltransferases, demethylases, and m^6^A modification binding proteins in degenerative discs from mice exposed to chronic inflammation. Our analysis revealed a notable increase in ALKBH5 expression, prompting us to delve into the impact of ALKBH5-mediated m^6^A demethylation in lipopolysaccharide (LPS)-treated nucleus pulposus (NP) cells and in mice with chronic inflammation-induced IDD. Our findings unveil that the inflammatory microenvironment triggers ALKBH5 expression, thereby augmenting m^6^A demethylation within the 3′-UTR of *Runx2* mRNA, culminating in heightened stability of *Runx2* mRNA and subsequent upregulation of *ADAMTSs* and *MMPs*. Furthermore, we conducted in vivo assessments to elucidate the involvement of Runx2, MMP1a, ADAMTS10, ALKBH5, and YTHDF1 in IDD progression. Together, our results establish a potential link between ALKBH5-mediated m^6^A modification of *Runx2* mRNA and the dysregulation of matrix-degrading enzymes in IDD development following exposure to chronic inflammation.

## Materials and methods

### Chronic inflammation animals

C57BL/6 mice were purchased from Charles River Laboratories (Beijing, China) and were housed in a specific pathogen-free (SPF) animal facility under a 12-hour light-dark cycle. ALKBH5 knockout (ALKBH5^KO^) and YTHDF1 overexpression (YTHDF1^OE^) mice were ordered from Cyagen (Suzhou, China). Eight weeks old male mice (*n* = 8, body weights ranging from 22 to 25 g) were intraperitoneally injected with 20 µg/kg LPS (Sigma-Aldrich, Shanghai, China; #L4516) in phosphate-buffered saline (PBS) solution (pH 7.4) (Thermo Fisher, Shanghai, China; #AM9624) once a week. Another group of mice (*n* = 8) received PBS injections at the same time points and were used as controls. After administration of LPS and PBS for 24 weeks, mice were anesthetized using an isoflurane inhalant and subjected to evaluate lumbar disc degeneration through X-ray imaging, following a previously established protocol [18]. Mice were subsequently euthanized by CO_2_ inhalation and blood and lumbar discs were immediately collected. All animal experiments were performed following a protocol approved by the Institutional Animal Care and Use Committee (IACUC) of Kunming Medical University.

### Administration of recombinant Runx2, MMP1a, and ADAMTS proteins near lumbar discs

Eight weeks old C57BL/6 mice (body weights ranging from 22 to 25 g) were randomly divided into 8 groups (*n* = 8 for each group). Near the lumbar discs, mice received subcutaneous injections of PBS (control), recombinant Runx2 protein (Applied Biological Materials, Zhenjiang, Jiangsu, China; #42,890,045; 20 mg/kg), MMP1a protein (Applied Biological Materials; #30,241,044; 20 mg/kg), ADAMTS10 protein (R&D Systems, Inc., Shanghai, China; #946-AD-020; 20 mg/kg), MMP1a and ADAMTS10 proteins (20 mg/kg each), Runx2 protein (20 mg/kg) combined with 10 mg/kg CADD522 (MedChemExpress LLC, Monmouth Junction, NJ, USA; #HY-107,999), Runx2 protein (20 mg/kg) with MMP1a (20 mg/kg) and 10 mg/kg CADD522, or Runx2 protein (20 mg/kg) with ADAMTS10 (20 mg/kg) and 10 mg/kg CADD522. Injections were administered once weekly for 24 weeks. At the study’s conclusion, mice underwent lumbar disc degeneration evaluation through X-ray imaging, followed by euthanasia via CO_2_ inhalation. Blood and lumbar discs were collected immediately. All experiments were approved by the Institutional Animal Care and Use Committee (IACUC) of Kunming Medical University.

### Isolation and culture of primary NP cells

Primary NP cells were isolated from lumbar IVDs of C57BL/6 mice injected with either PBS or 20 µg/kg LPS, following a previously established protocol [27]. Lumbar IVDs were thoroughly rinsed with Hanks’ Balanced Salt solution (Sigma-Aldrich; #H8264) under sterile conditions to remove blood cells. Subsequently, the NP tissues were dissected into 1–2 mm^3^ fragments and digested using trypsin/EDTA solution (Thermo Fisher; #R001100) at 37 °C to eliminate fibrous tissues. The digested tissue was then incubated with 0.1% collagenase I (Thermo Fisher; #17,018,029) and 0.5 × trypsin/EDTA solution at 37 °C for 1 h in a shaking incubator set at 200 *g*. Following digestion, the released cells were harvested by centrifugation at 1000 *g* for 10 min and resuspended in 5 mL of α-MEM (Thermo Fisher; #12,571,063) supplemented with 10% fetal bovine serum (FBS) (Thermo Fisher; #16,000,044). The cells were cultured in Dulbecco’s Modified Eagle Medium (DMEM) with 10% FBS at 37 °C in a 5% CO_2_ incubator, and cells at passage 5 were utilized for subsequent experiments. Primary cells isolated from mice injected with PBS and LPS were designated as normal NPs (NNPs) and LPS-exposed NPs (LENPs), respectively.

### Cell transfection

Depletion and overexpression of ALKBH5 and Runx2 in primary NP cells were achieved using lentiviral vectors (Table S1) and plasmids (Table S2), respectively. The lipofectamine 3000 reagent (Thermo Fisher; #L3000001) was employed to transfect the vectors into the cells following the manufacturer’s instructions. Stable expressing and knockdown cells were selected by culturing in DMEM supplemented with 10% FBS and 1 µg/mL puromycin (Thermo Fisher; #A1113802) for 72 h post-infection.

### Measurement of proinflammatory cytokines by enzyme linked immunosorbent assay (ELISA)

Blood samples were centrifuged at 1000 × g for 10 min to separate serum. The supernatant serum samples were collected and subjected to ELISA assays using different kits for proinflammatory cytokines, including IL-1β (Invitrogen, Shanghai, China; #BMS6002), IL-6 (Invitrogen; #KMC0061), and TNF-α (Invitrogen; #BMS607-2HS). The ELISA assay procedures were performed according to the manufacturer’s instructions. For cell culture supernatants, NNP1/2 cells at 80% confluence were treated with varying concentrations (0, 20, 40, and 80 ng/mL) of LPS for 6 h. Subsequently, the supernatants from the cell cultures were collected following the LPS treatments. The concentrations of IL-1β, IL-6, and TNF-α in the supernatants were determined using the same ELISA protocol as described for serum samples.

### Total RNA isolation and real time-quantitative PCR (RT-qPCR)

RNA extraction was carried out using Trizol (Thermo Fisher; #15,596,026). One microgram of RNA was reverse-transcribed into complementary DNA (cDNA) using the LunaScript RT SuperMix Kit (New England Biolabs, Shanghai, China; #M3010) following the manufacturer’s instructions. The resulting cDNA samples were diluted 50-fold and subjected to RT-qPCR analyses to determine gene expression levels, using the PowerTrack SYBR Green Master Mix (Thermo Fisher; #A46012) and primers listed in Table S3. Beta-Actin (β-Actin) was used as the reference gene. The relative gene expression levels were calculated by the 2^−ΔΔCt^ method.

### Western blotting

Total proteins were extracted from lumbar discs and cultured cells using radioimmunoprecipitation assay (RIPA) buffer (Thermo Fisher; #89,900) in the presence of 1 × Protease and Phosphatase Inhibitor Cocktail (Thermo Fisher; #78,440). Equal amounts (50 µg) of proteins were loaded onto a 10% sodium dodecyl-sulfate polyacrylamide gel electrophoresis (SDS-PAGE) for separation. Proteins were transferred to polyvinylidene difluoride (PVDF) membranes (Thermo Fisher; #88,518), followed by blocking membranes with 5% skim milk (Thermo Fisher; #LP0033B). The membranes were incubated with primary antibodies (Table S4) overnight at 4 °C, followed by secondary antibodies (Table S4) for 1 h at room temperature. Protein bands were visualized using an enhanced chemiluminescence (ECL) kit (Thermo Fisher; #32,106). Quantification of protein levels was conducted using Image J software (National Institutes of Health, USA), and the relative protein expression levels were normalized to their corresponding loading controls.

### Methylated RNA immunoprecipitation (MeRIP) and RT-qPCR

Total RNA was extracted from lumbar discs and cultured cells with different treatments. Genomic DNA contamination was eliminated from the RNA samples through DNase I treatment (Thermo Fisher; #EN0521) at 37 °C for 10 min. The resulting RNA samples were subjected to mRNA purification and fragmentation, followed by immunoprecipitation using an anti-m^6^A primary antibody (Merck Millipore; Billerica, MA, USA; #ABE572) and a Magna MeRI m^6^A kit (Merck Millipore; #17–10,499) according to the manufacturer’s guidelines. The enriched m^6^A-modified mRNA was detected by RT-qPCR using primers listed in Table S5.

### Determination of RNA stability

RNA stability was performed following a previously established protocol [28]. Briefly, cells at 80% confluence were treated with 5 µg/mL actinomycin D (Sigma-Aldrich; #A1410) at 37 °C for 0, 3, and 6 h. After treatment, cells were washed twice with PBS and total RNA was isolated, followed by DNase I digestion. RNA degradation rate was assessed following the previously described protocol [28].

### Histological staining

The histological structure of lumbar IVDs was performed using a previously established staining method [18]. Briefly, the L1/L2 lumbar IVDs from different groups of mice were fixed with 10% Neutral Buffered Formalin and decalcified using a mild decalcifier-solution for 24 and 72 h, respectively. The samples were then dehydrated with ethanol, embedded in paraffin, and sectioned into 10 μm thick slices. After deparaffinization and rehydration, the sections were stained using a series of solutions: 1% alcian blue, 0.02% fast green, 1.3% picric acid, and 1.0% Safranin O. The slides were then rinsed, dehydrated, cleared, and coverslipped before being photographed using an inverted TE 2000 wide-field microscope system. Histological images were scored according to a previously established standard [29]. For annulus fibrosus (AF) scoring, grades were assigned as follows: 0 for normal appearance, 1 for mild serpentine shape, 2 for moderate serpentine shape (slightly bulging beyond the endplate edge), 3 for severe serpentine shape (clearly bulging beyond the endplate edge), 4 for severe serpentine shape with rupture, and 5 for indistinct features. For nucleus pulposus (NP) scoring, grades were assigned as follows: 0 for normal appearance, 1 for condensed structure, 2 for presence of chondrocyte-like cells with residual NP matrix, 3 for widespread presence of chondrocyte-like cells, 4 for mild replacement by fibrous cartilaginous tissue, and 5 for moderate or severe replacement by fibrous cartilaginous tissue.

### Chromatin immunoprecipitation (ChIP) assay

For ChIP assay in lumbar discs, fresh tissues (0.1 g) were chopped into 1 mm^3^ pieces with two razor blades. Chopped pieces were crosslinked using 1.5% formaldehyde (Sigma-Aldrich; #47,608) for 15 min. For ChIP assay in cultured cells, approximately 5 × 10^7^ cells were crosslinked using 1% formaldehyde for 10 min. The crosslinking reaction in both tissues and cells were terminated by adding glycine to a final concentration of 125 mM with shaking (200 rpm) for 5 min at room temperature. Subsequently, the tissues and cells were lysed using ChIP lysis buffer (5 mM PIPES pH 8.0, 85 mM KCl, 0.5% NP-40, and 1 × protease inhibitor cocktail) and the chromatin was sonicated to obtain fragments with an average length of 300–500 bp. After centrifugation to remove cell debris, the supernatants were incubated with anti-Runx2 and IgG-coated protein A agarose at 4 °C for 2 h for immunoprecipitation. The purified input and output DNAs were used as templates for ChIP-RT-qPCR assays, employing primers listed in Table S6.

### Human IVD sample collection

Non-degenerated IVDs (*n* = 10) were sourced from deceased donors who, prior to their demise in fatal accidents, had willingly signed organ donation consent forms. Blood tests conducted on these donors showed no indications of notable inflammation. Degenerated IVDs (*n* = 10) were procured from patients necessitating intervertebral disc replacement surgery, all of whom presented chronic inflammation linked to IDD. All donors gave informed consent for the experimental procedures. The basic information of all participants was summarized in Table S7.

### Statistical analysis

All experiments were conducted in triplicate to ensure the reliability of the data. The results are presented as the mean ± standard deviation (SD). Statistical analyses were performed using the one-way analysis of variance (ANOVA) test with Tukey’s post-hoc test in the Statistical Package for the Social Sciences (SPSS) software (IBM, NY, USA; version 18) to compare between groups. A *P*-value of less than 0.05 was considered to be statistically significant. We denoted the level of significance with asterisks, where one asterisk (*) represents *P* < 0.05, two asterisks (**) represent *P* < 0.01, three asterisks (***) represent *P* < 0.001, and four asterisks (****) represent *P* < 0.0001.

## Results

### Elevated expression of ALKBH5 and reduced YTHDF1 were observed in degenerative IVDs from mice with chronic inflammation

**Fig. 1:**
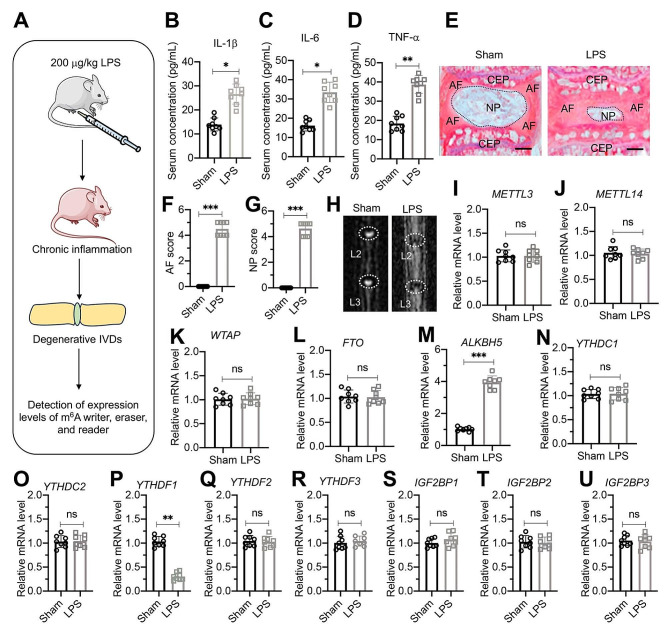
Altered expression of ALKBH5 and YTHDF1 was observed in degenerative IVDs from LPS-challenged mice (**A**) Experimental design depicting the study methodology. **(B-D)** Circulating concentrations of IL-1β (**B**), IL-6 (**C**), and TNF-α (**D**) in mice injected with PBS (sham) or LPS (*n* = 8 for each group). (**E**) Representative histological images of lumbar IVDs. The L1/L2 lumbar IVDs from sham and LPS groups of mice were stained with safranin O and fast green. The locations of NP, AF, and cartilage endplate (CEP) were labeled. Bars = 200 μm. **(F** and **G)** Quantified AF and NP scores in histological images of lumbar IVDs as depicted in (E). (**H**) Representative micro-CT images of lumbar IVDs (L2/L3) from sham and LPS groups. **(I-U)** The mRNA expression levels of *METTL3*(**I**), *METTL14*(**J**), *WTAP*(**K**), *FTO*(**L**), *ALKBH5*(**M**), *YTHDC1*(**N**), *YTHDC2*(**O**), *YTHDF1*(**P**), *YTHDF2*(**Q**), *YTHDF3*(**R**), *IGF2BP1*(**S**), *IGF2BP2*(**T**), and *IGF2BP3*(**U**) in IVDs from sham and LPS groups (*n* = 8 for each group). *P < 0.05; **P < 0.01; ***P < 0.001; ns represents no significant difference

To explore the potential alterations in the expression levels of m^6^A methyltransferases, RNA demethylases, and m^6^A modification binding proteins in the pathogenesis of IDD induced by chronic inflammation, we established a chronic inflammation mouse model by weekly administration of a low dose of LPS (20 µg/kg) (Fig. 1A). Over the 24-week period of LPS administration, we observed a mild increase in serum IL-1β, IL-6, and TNF-α levels (Fig. 1B and D). Moreover, the low-dose LPS regimen led to degenerative lumbar IVDs (Fig. 1E). Quantification of AF and NP scores further revealed severe degeneration of both AF and NP tissues following LPS administration (Fig. 1F and G). Micro–computed tomography (micro-CT) results corroborated the promotion of lumbar disc degeneration by chronic inflammation (Fig. 1H).

Subsequent evaluation of mRNA and protein expression levels included METTL3, METTL14, WTAP, FTO, ALKBH5, YTHDC1, YTHDC2, YTHDF1, YTHDF2, YTHDF3, IGF2BP1, IGF2BP2, and IGF2BP3 in lumbar IVDs from sham (injection with PBS) and LPS-administered mice. Among these genes and their corresponding proteins, we observed an increase in ALKBH5 mRNA/protein expression and a decrease in YTHDF1 mRNA/protein levels (Fig. 1I and U and S1). However, the expression levels of the other 11 genes and their encoded proteins remained unchanged in degenerative discs (Fig. 1I and U and S1).

### Elevated expression of ALKBH5 and reduced YTHDF1 were observed in LPS-treated and proinflammatory cytokine-treated NP cells

To investigate the influence of the inflammatory microenvironment on ALKBH5 and YTHDF1 expression levels, primary NNP cells were treated with varying doses of LPS (0, 20, 40, and 80 ng/mL), and their expression levels were determined. Additionally, mRNA and protein levels of METTL3 and IGF2BP1 were examined as controls. Consistent with the in vivo findings, LPS treatments did not alter the mRNA levels of *METTL3* and *IGF2BP1* but resulted in a dose-dependent increase in *ALKBH5* mRNA and a dose-dependent decrease in *YTHDF1* mRNA levels (Figures S2A-S2D). Protein levels of METTL3, IGF2BP1, ALKBH5, and YTHDF1 mirrored these mRNA level changes (Figures S2E and S2F). Furthermore, concentrations of IL-1β, IL-6, and TNF-α in cell culture supernatants increased dose-dependently following LPS exposure (Figure S3).

To assess the direct effect of individual proinflammatory cytokines on METTL3, IGF2BP1, ALKBH5, and YTHDF1 expression, NNP cells were treated with varying doses (0, 20, 40, and 60 ng/mL) of IL-1β, IL-6, and TNF-α. RT-qPCR results indicated that mRNA levels of *METTL3* and *IGF2BP1* remained unchanged following treatments with these cytokines (Figures S4A, S4B, S5A, S5B, S6A, and S6B). Conversely, *ALKBH5* mRNA levels increased dose-dependently, while *YTHDF1* mRNA levels decreased dose-dependently following treatments with IL-1β, IL-6, and TNF-α (Figures S4C, S4D, S5C, S5D, S6C, and S6D). Protein levels of METTL3 and IGF2BP1 remained unchanged, whereas protein levels of ALKBH5 increased dose-dependently and YTHDF1 decreased dose-dependently following treatments with IL-1β, IL-6, and TNF-α (Figures S4E, S4F, S5D, S5F, S6D, and S6F). These results indicate that the inflammatory microenvironment induced by LPS and proinflammatory cytokines can regulate the expression of ALKBH5 and YTHDF1.

### Expression levels of *Runx2*and multiple *ADAMTS* and *MMP* genes were dependent on ALKBH5

**Fig. 2:**
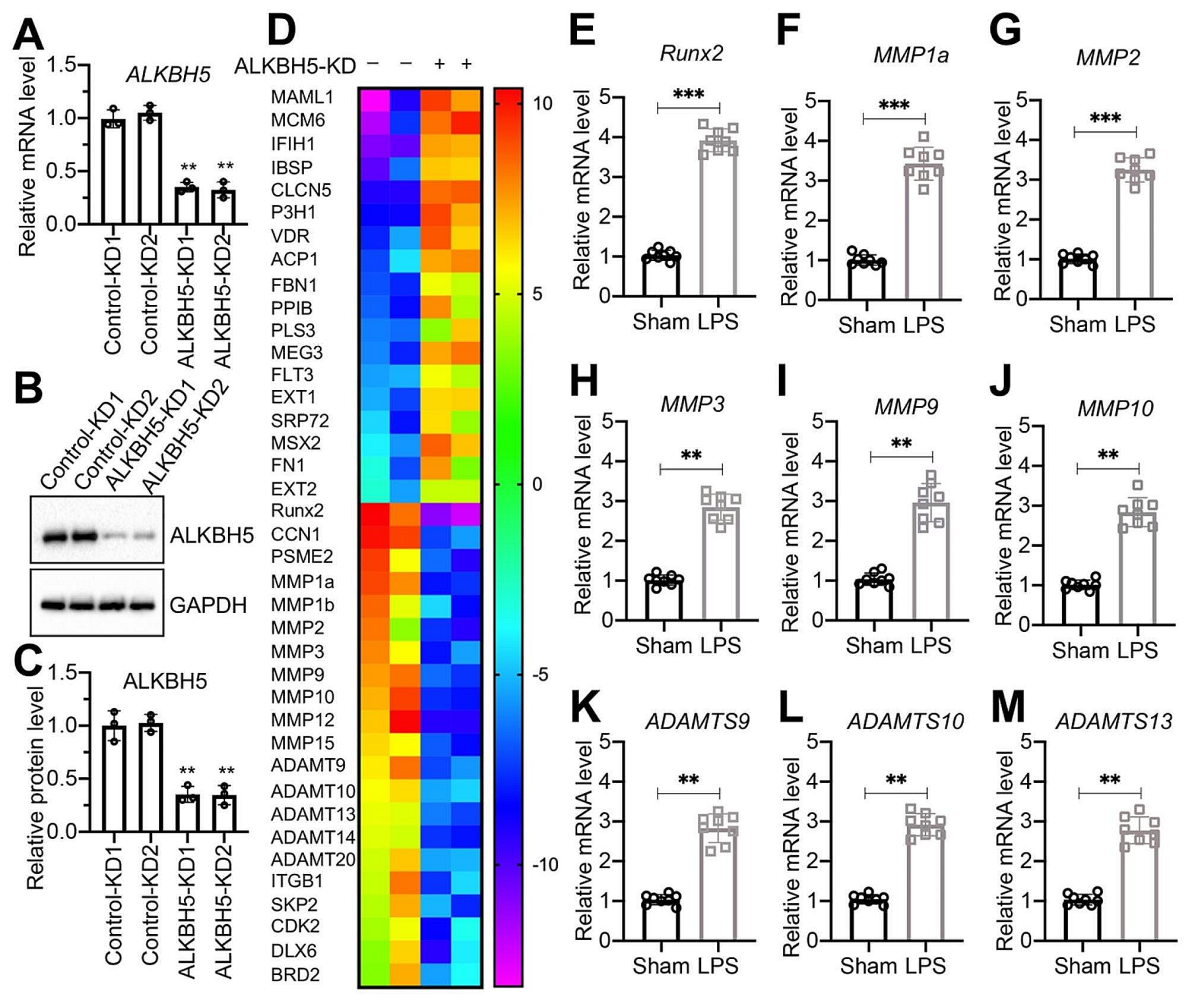
Identification of ALKBH5-dependent differentially expressed genes and validation of*MMP* and *ADAMTS* gene expression in IVDs(**A**)*ALKBH5* mRNA levels in Control-KD (#1 and #2) and ALKBH5-KD (#1 and #2) cell lines. (**B**) Western blotting results showing ALKBH5 protein levels. (**C**) Quantification of ALKBH5 protein levels. (**D**) Heatmap illustrating the results of microarray analysis. RNA samples from Control-KD and ALKBH5-KD cell lines were subjected to microarray analysis, revealing dysregulated genes displayed in the heatmap. **(E-M)** mRNA levels of *MMP* and *ADAMTS* genes in IVDs. RT-qPCR analysis was performed using RNA samples isolated from L1/L2 IVDs of sham and LPS groups of mice (*n* = 8 for each group) to investigate the mRNA levels of *Runx2*(**E**), *MMP1a*(**F**), *MMP2*(**G**), *MMP3*(**H**), *MMP9*(**I**), *MMP10*(**J**), *ADAMTS9*(**K**), *ADAMTS10*(**L**), and *ADAMTS13*(**M**). **P < 0.01; ***P < 0.001

To identify genes regulated by ALKBH5-dependent m^6^A modification, we established stable ALKBH5-depleted cells in primary LENP-1 background (Fig. 2A and C). Subsequently, a microarray analysis was conducted to identify genes whose expression was influenced by ALKBH5. The analysis unveiled 19 consistently upregulated genes in both ALKBH5-KD1 and ALKBH5-KD2 cells (Fig. 2D and Table S8). Additionally, 21 genes displayed consistent downregulation in both ALKBH5-KD1 and ALKBH5-KD2 cells (Fig. 2D and Table S8). Notably, among these 21 downregulated genes were *Runx2*, the pivotal transcription factor in bone development and differentiation, alongside two classes of proteases (MMPs and ADAMTSs), encompassing *MMP1a*, *MMP1b*, *MMP2*, *MMP3*, *MMP9*, *MMP10*, *MMP12*, *MMP15*, *ADAMTS9*, *ADAMTS10*, *ADAMTS13*, *ADAMTS14*, and *ADAMTS20* (Fig. 2D and Table S8). To corroborate the significance of these findings, we evaluated the mRNA levels of several representative genes in lumbar IVDs obtained from mice subjected to either sham or LPS administration (*n* = 8 for each group). Remarkably, the expression levels of *Runx2*, *MMP1a/2/3/9/10*, and *ADAMTS9/10/13* were markedly elevated in degenerative IVDs from LPS-administered mice (Fig. 2E and M). Furthermore, we observed a dose-dependent increase in these genes in LPS-treated NNP-1 and NNP-2 cells (Figure S7).

### Both *MMP1a/2/3/9/10* and *ADAMTS9/10/13* were downstream target genes of Runx2

**Fig. 3:**
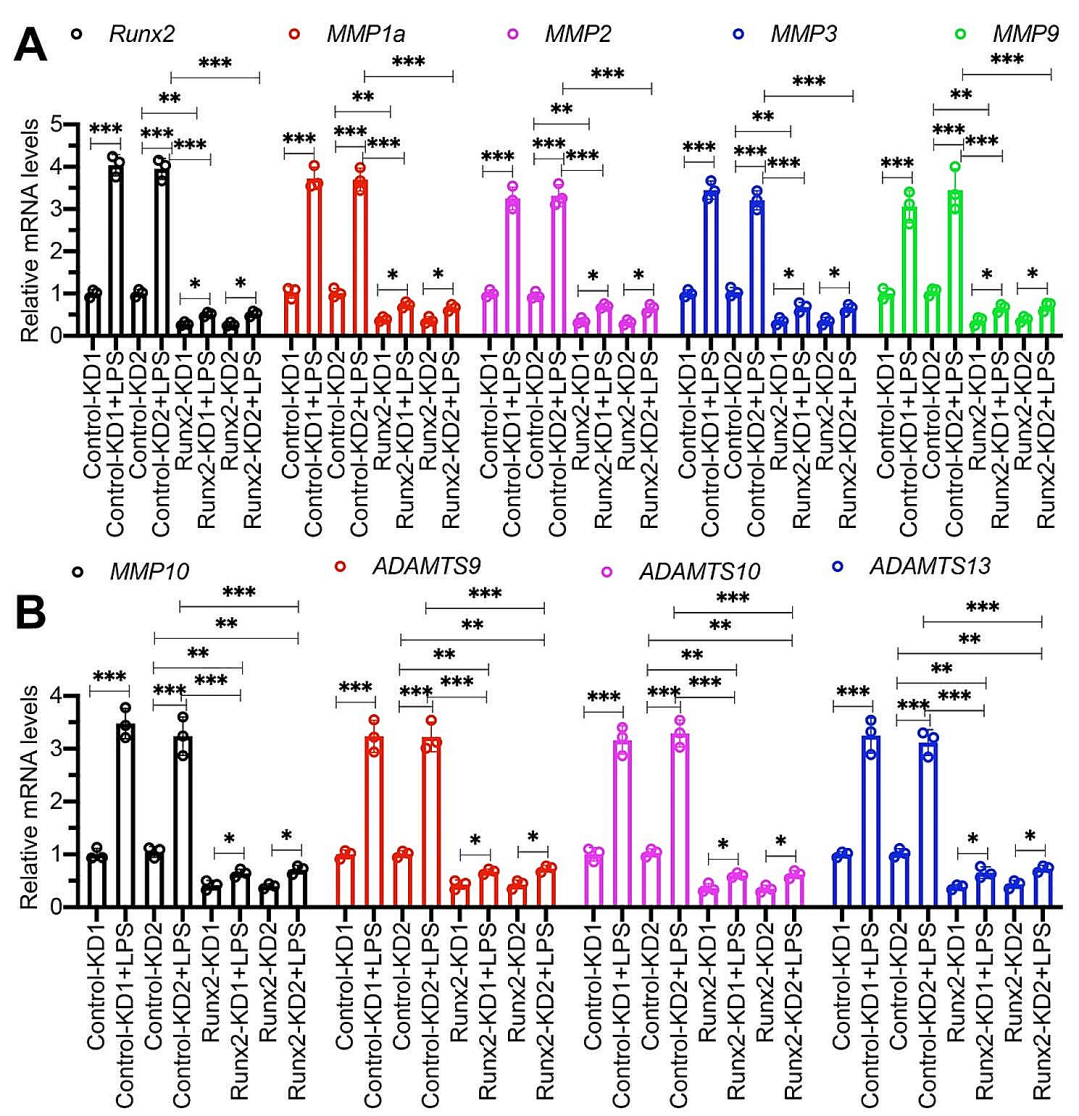
Effect of LPS on the expression levels of *MMPs* and*ADAMTSs* in Runx2-KD cells The Control-KD (#1 and #2) and Runx2-KD (#1 and #2) cell lines were treated with 80 ng/mL LPS for 6 h, followed by RNA isolation and RT-qPCR assays to measure mRNA levels of *Runx2/MMP1a/MMP2/MMP3/MMP9*(**A**) and *MMP10/ADAMTS9/ADAMTS10/ADAMTS13*(**B**). *P < 0.05; **P < 0.01; ***P < 0.001

**Fig. 4:**
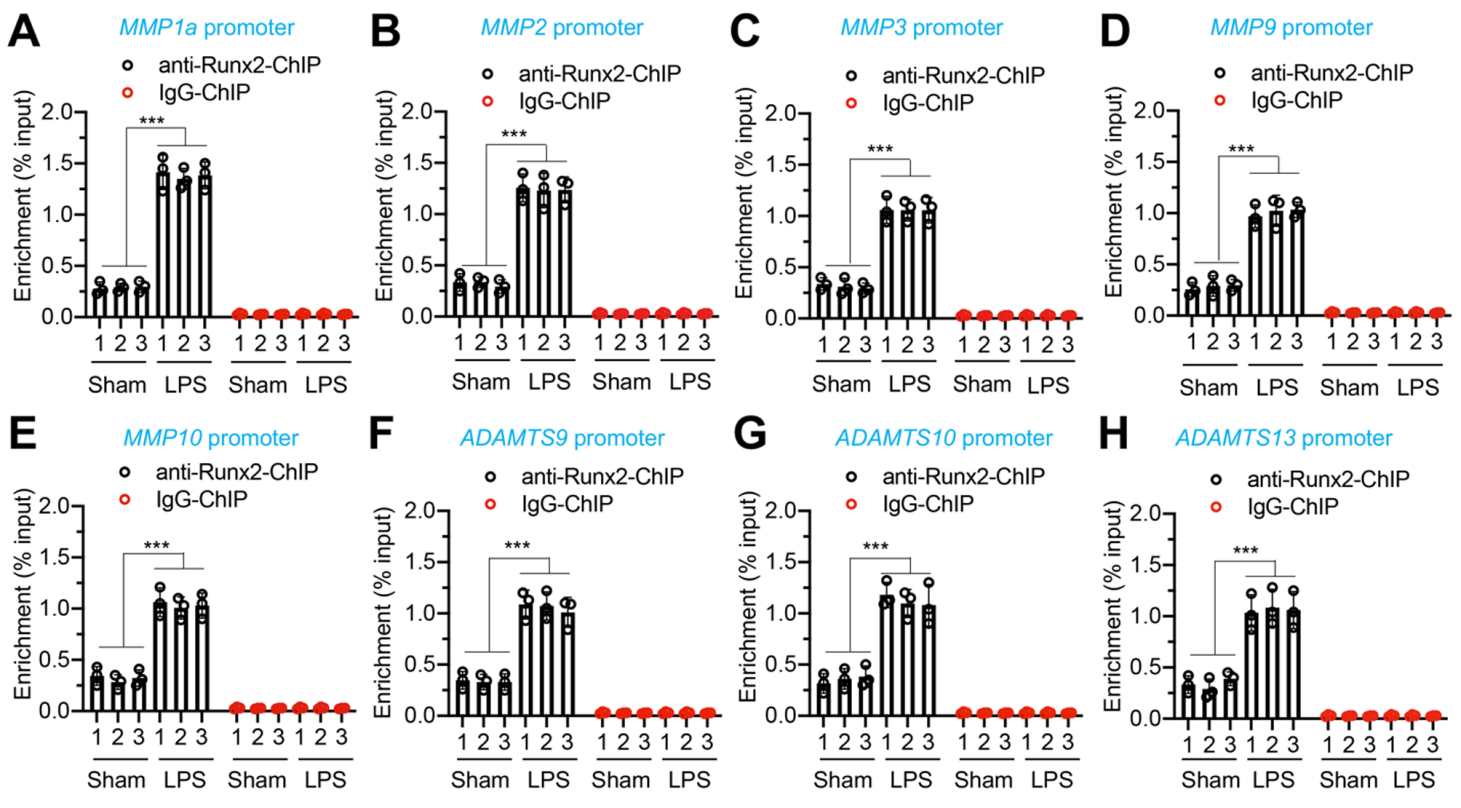
The binding of Runx2 on the promoters of *MMP1a/2/3/9/10*and *ADAMTS9/10/13* was significantly increased in degenerative IVDs Three representative IVDs from sham and LPS groups of mice were used for ChIP assay with anti-Runx2- or IgG-coated protein G agarose. Input and output DNA samples were subjected to RT-qPCR analyses to measure the enrichment of Runx2 on the promoters of *MMP1a*(**A**), *MMP2*(**B**), *MMP3*(**C**), *MMP9*(**D**), *MMP10*(**E**), *ADAMTS9*(**F**), *ADAMTS10*(**G**), and *ADAMTS13*(**H**). ***P < 0.001

Several *MMP* genes, including *MMP2*, *MMP9*, and *MMP13*, as well as *ADAMTS* genes such as *ADAMTS4* and *ADAMTS5*, have been identified as being regulated by Runx2 [18, 30, 31]. We conducted a search using the consensus sequence (TGTGGT) of Runx2 within the promoters of *MMP1a*, *MMP1b*, *MMP2*, *MMP3*, *MMP9*, *MMP10*, *MMP12*, *MMP15*, *ADAMTS9, ADAMTS10*, *ADAMTS13*, *ADAMTS14*, and A*DAMTS20*. Interestingly, we found that all thirteen gene promoters contained at least one Runx2 binding site (Figure S8). To further investigate the functional role of Runx2 in regulation of these *MMPs* and *ADAMTSs*, we established stable Runx2-depleted cells in primary LENP-1 cells (Figure S9). As a result of the numerous *MMPs* and *ADAMTSs* genes, we selectively analyzed the expression of *MMP1a/2/3/9/10* and *ADAMTS9/10/13*. Our findings indicate a marked reduction in the expression levels of these 7 genes within the Runx2-KD cells (Fig. 3). The induction of *MMP1a/2/3/9/10* and *ADAMTS9/10/13* by 80 ng/mL LPS was significantly repressed in Runx2-KD cells (Fig. 3). ChIP RT-qPCR analyses revealed a significant enrichment of Runx2 occupancy on the promoters of *MMP1a/2/3/9/10* and *ADAMTS9/10/13* in degenerative IVDs from LPS-administered mice compared to sham mice (Fig. 4). Furthermore, the depletion of Runx2 in primary LENP-1 cells resulted in a significant decrease in its binding to the promoters of *MMP1a/2/3/9/10* and *ADAMTS9/10/13* (Figure S10). The LPS treatments in Runx2-KD cells resulted in a slight increase in the bindings of Runx2 on *MMP* and *ADAMTS* promoters, which was significantly lower compared to that observed in Control-KD cells (Figure S10). These findings strongly suggest that *MMP1a/2/3/9/10* and *ADAMTS9/10/13* are downstream target genes of Runx2.

### Administration of recombinant Runx2, MMP1a, and ADAMTS10 in mice resulted in degeneration of lumbar discs

**Fig. 5:**
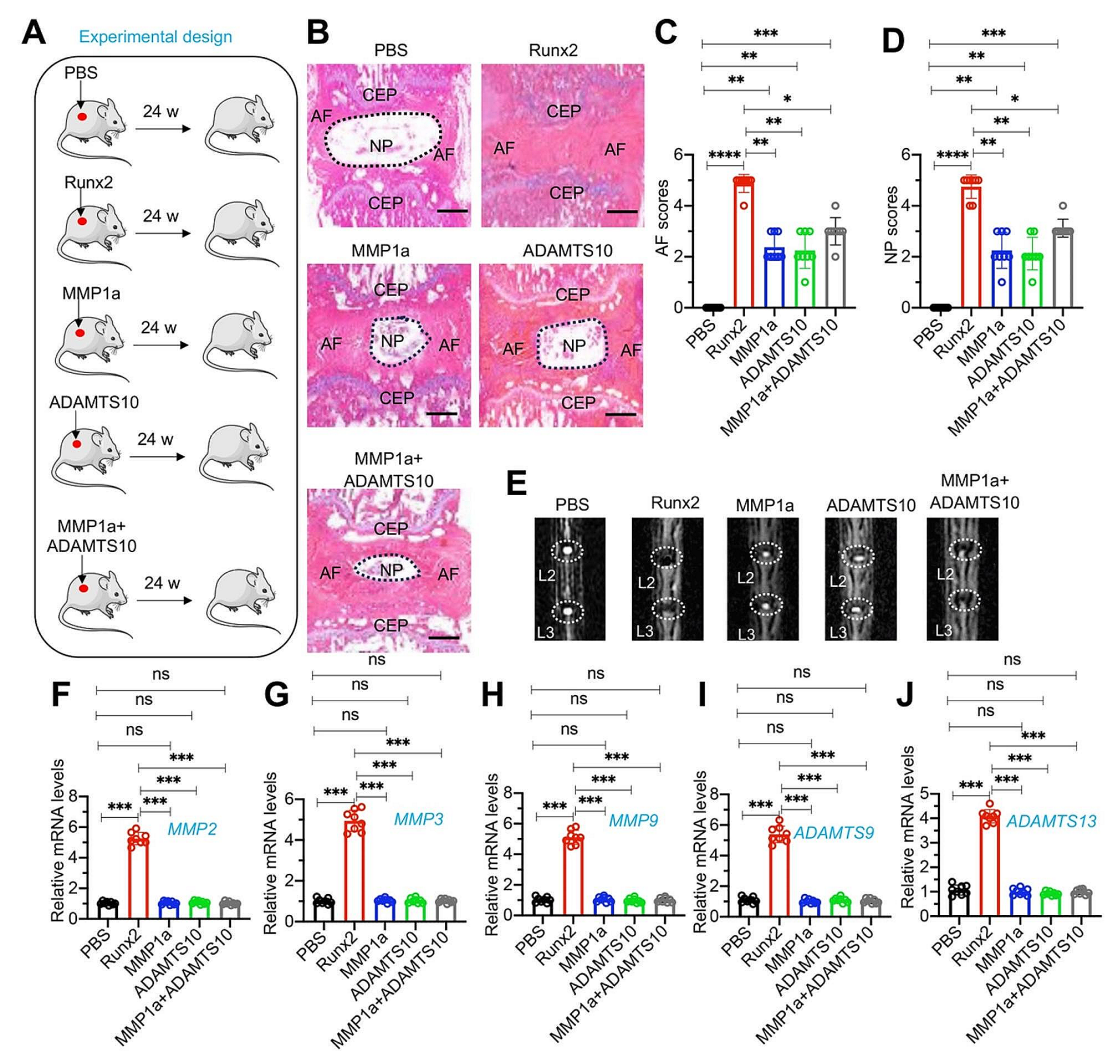
Administration of recombinant Runx2, MMP1a and ADAMTS10 in mice ed to varying degrees of IDD(**A**) Experimental design depicting the study methodology. (**B**) Representative histological images of lumbar IVDs. The L1/L2 lumbar IVDs from PBS, Runx2, MMP1a, ADAMTS10, and MMP1a + ADAMTS10 groups of mice were stained with safranin O and fast green. The locations of NP, AF, and CEP were labeled. Bars = 200 μm. **(C** and **D)** Quantified AF and NP scores in histological images of lumbar IVDs as depicted in (B). (**E**) Representative micro-CT images of lumbar IVDs (L2/L3) from PBS, Runx2, MMP1a, ADAMTS10, and MMP1a + ADAMTS10 groups of mice. **(F-J)** The mRNA expression levels of *MMP2*(**F**), *MMP3*(**G**), *MMP9*(**H**), *ADAMTS9*(**I**), and *ADAMTS13*(**J**) in IVDs from PBS, Runx2, MMP1a, ADAMTS10, and MMP1a + ADAMTS10 groups of mice (*n* = 8 for each group). *P < 0.05; **P < 0.01; ***P < 0.001; ns represents no significant difference

Our in vitro findings imply a crucial role for Runx2 in the regulation of IDD, particularly through its influence on the expression of *MMPs* and *ADAMTSs*. To further explore the in vivo role, we assessed the expression levels of Runx2 in both sham and LPS-injected mice. Notably, there was a significant upregulation of Runx2 at the mRNA and protein levels in the LPS-treated mice (Figure S11). We extended our investigation to determine if the lumbar disc degeneration observed was a direct consequence of Runx2, as well as the involvement of MMPs and ADAMTSs. To this end, we administered subcutaneous injections of recombinant Runx2 protein adjacent to the lumbar discs in C57BL/6 mice. We also performed parallel injections with representative proteins from the MMP and ADAMTS families—specifically MMP1a and ADAMTS10—at the same anatomical site, in addition to a co-injection of MMP1a + ADAMTS10 (Fig. 5A). Our results demonstrated that PBS did not induce degeneration of the lumbar discs. In contrast, Runx2 injection led to complete disc degeneration. Individual injections of MMP1a or ADAMTS10 resulted in a spectrum of degeneration ranging from mild to moderate (Fig. 5B and E). Moreover, co-injection of MMP1a + ADAMTS10 exacerbated the degenerative process, causing a more severe degeneration than either protein alone (Fig. 5B and E).

To delineate the molecular mechanisms underpinning these observations, we profiled the expression of additional *MMPs* and *ADAMTSs*, including *MMP2*, *MMP3*, *MMP9*, *ADAMTS9*, and *ADAMTS13*, in the lumbar discs of all five experimental groups. A significant increase in the expression of these genes was exclusive to the Runx2-injected group, with no significant changes detected in the other groups (Fig. 5F and J). We further examined the expression of a set of known Runx2 target genes—*COL1A1* (Collagen, type I, alpha 1), *ALPL* (alkaline phosphatase, liver/bone/kidney), *BMP2* (bone morphogenetic protein 2), *IBSP* (integrin binding sialoprotein), *WNT10B* (Wnt family member 10B), and *FGFR1* (fibroblast growth factor receptor 1). Our examination revealed a slight increase in the expression levels of *COL1A1*, *ALPL*, *BMP2*, and *IBSP* in response to Runx2 injection, while there was no significant impact observed on *WNT10B* and *FGFR1* (Figure S12). Interestingly, neither *MMP1a* nor *ADAMTS10* exerted any noticeable influence on the expression of these genes (Figure S12). This suggests that Runx2 selectively upregulates *MMPs* and *ADAMTSs*, potentially reflecting a cell type-specific regulatory mechanism. Conversely, the individual injections of MMP1a and ADAMTS10 led to discernible lumbar disc degeneration but did not impact the expression levels of other members of the MMP and ADAMTS families.

### CADD522 impaired Runx2 function and protected lumbar disc from Runx2-mediated degeneration

**Fig. 6:**
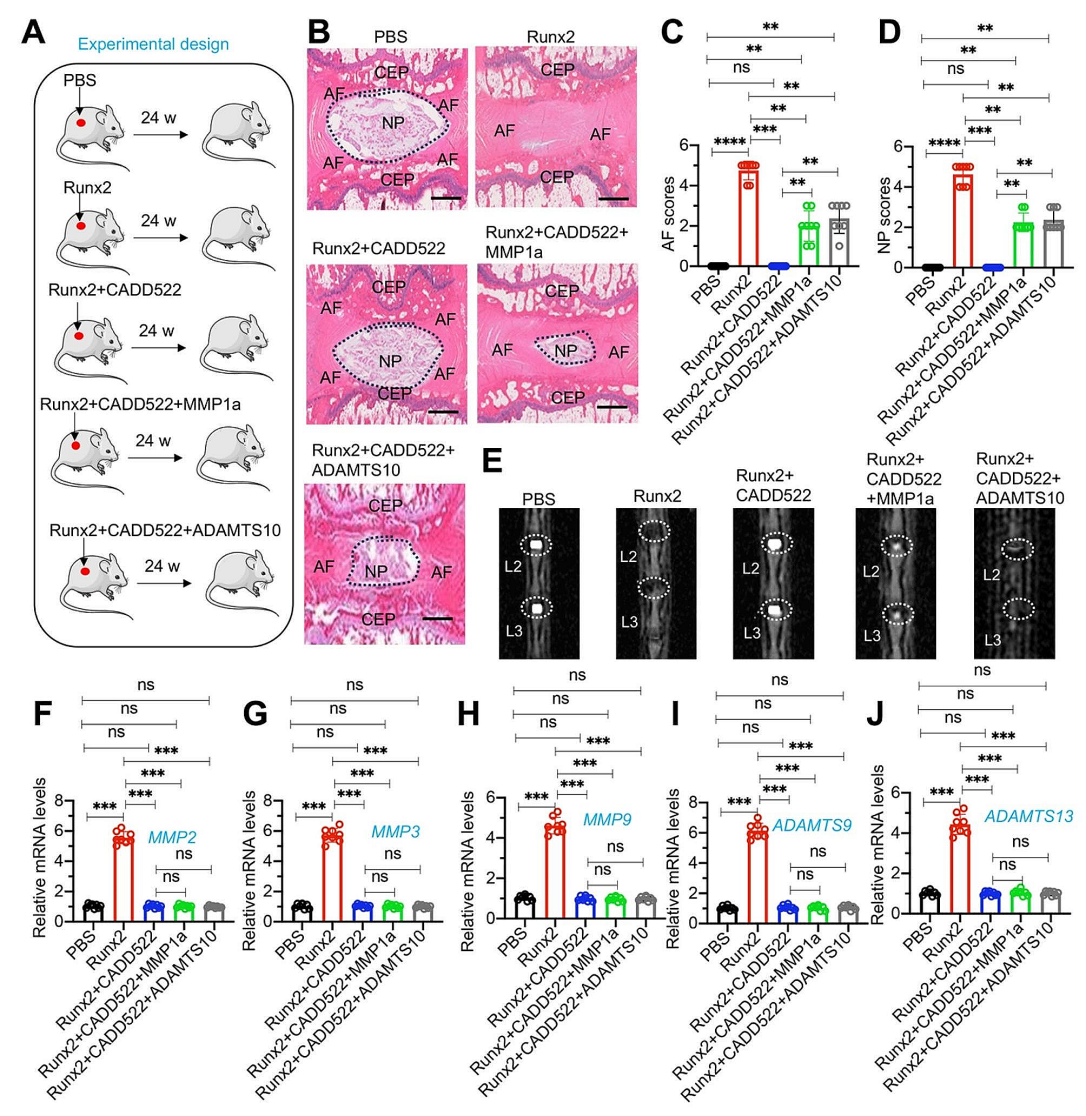
Administration of CADD522 in mice injected with recombinant Runx2 prevented the occurrence of IDD (**A**) Experimental design depicting the study methodology. (**B**) Representative histological images of lumbar IVDs. The L1/L2 lumbar IVDs from PBS, Runx2, Runx2 + CADD522, Runx2 + CADD522 + MMP1a, and Runx2 + CADD522 + ADAMTS10 groups of mice were stained with safranin O and fast green. The positions of NP, AF, and CEP were labeled. The positions of NP, AF, and CEP were labeled. Bars = 200 μm. **(C** and **D)** Quantified AF and NP scores in histological images of lumbar IVDs as depicted in (B). (**E**) Representative micro-CT images of lumbar IVDs (L2/L3) from PBS, Runx2, Runx2 + CADD522, Runx2 + CADD522 + MMP1a, and Runx2 + CADD522 + ADAMTS10 groups of mice. **(F-J)** The mRNA expression levels of *MMP2*(**F**), *MMP3*(**G**), *MMP9*(**H**), *ADAMTS9*(**I**), and *ADAMTS13*(**J**) in IVDs from PBS, Runx2, Runx2 + CADD522, Runx2 + CADD522 + MMP1a, and Runx2 + CADD522 + ADAMTS10 groups of mice (*n* = 8 for each group). **P < 0.01; ***P < 0.001; ns represents no significant difference

Given that Runx2 can promote the occurrence of IDD, we subsequently evaluated whether the Runx2 inhibitor CADD522 could mitigate this process. For this purpose, mice were randomly divided into five groups and received subcutaneous injections near the lumbar disc with either PBS, recombinant Runx2 protein, Runx2 + CADD522, Runx2 + CADD522 + MMP1a, or Runx2 + CADD522 + ADAMTS10 (Fig. 6A). The results indicated that injections of Runx2 + CADD522 did not lead to degeneration of the lumbar disc (Fig. 6B and E). Mice injected with Runx2 + CADD522 in combination with MMP1a or ADAMTS10 exhibited mild to moderate lumbar disc degeneration, similar to the outcomes observed with injections of MMP1a and ADAMTS10 alone (Fig. 6B and E). Analysis of the expression of *MMP2*, *MMP3*, *MMP9*, *ADAMTS9*, and *ADAMTS13* in the lumbar discs of these five groups revealed a significant upregulation of these genes only in the group injected with Runx2, with no significant differences noted in the other four groups (Fig. 6F and J). Moreover, the expression levels of additional Runx2 target genes (*COL1A1*, *ALPL*, *BMP2*, and *IBSP*) exhibited only a slight increase in the Runx2-injected group, with no statistically significant differences observed among the other four groups (Figure S13). Notably, there were no alterations detected in the expression levels of *WNT10B* and *FGFR1* across all five groups (Figure S13). These findings explicitly demonstrate that inhibiting Runx2 can suppress the occurrence of IDD. They also reaffirm that MMP1a and ADAMTS10 can cause lumbar disc degeneration to a certain extent.

### ALKBH5 mediated m ^6^A demethylation of *Runx2* mRNA to maintain its stability and cause its overexpression

**Fig. 7:**
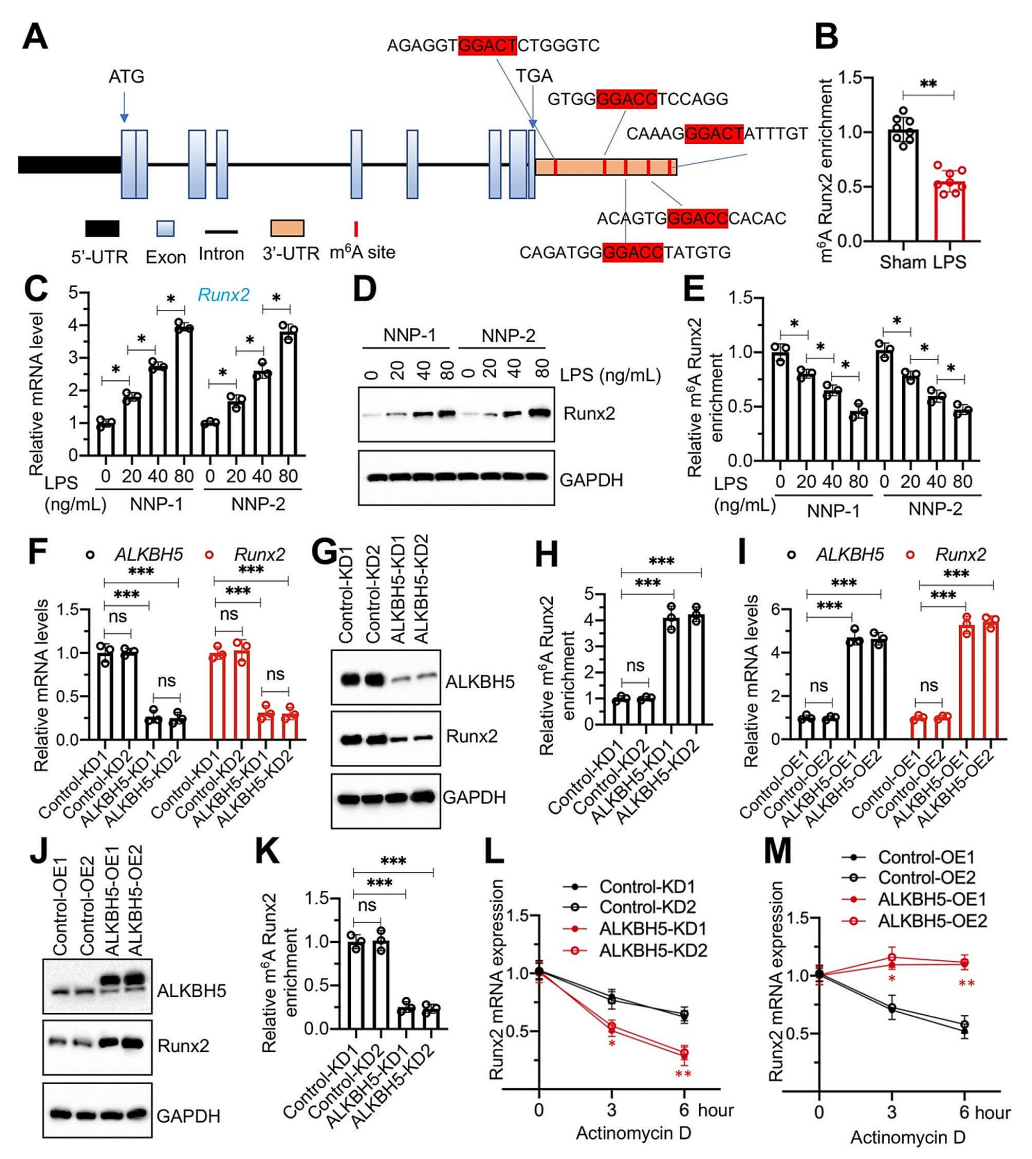
ALKBH5 mediated m^6^ A demethylation of *Runx2* mRNA (**A**) Schematic representation showing the positions of m^6^A motifs in the 3’-UTR of *Runx2* mRNA. (**B**) m^6^A methylation of *Runx2* mRNA in IVDs from both sham and LPS-administrated mice by MeRIP-qPCR assays. **(C** and **D)** Runx2 mRNA and protein levels in NNP cells (#1 and #2) treated with varying doses (0, 20, 40, and 80 ng/mL) of LPS. (**C**) *Runx2* mRNA levels. (**D**) Runx2 protein levels. (**E**) m^6^A methylation of *Runx2* mRNA in LPS-treated NNP cells. (**F**)*Runx2* mRNA levels in ALKBH5-KD cells (#1 and #2) under LENP-1 background. (**G**) Protein levels of Runx2 in ALKBH5-KD cells under LENP-1 background. (**H**) m^6^A methylation of *Runx2* mRNA in ALKBH5-KD cells under LENP-1 background. (**I**)*Runx2* mRNA levels in ALKBH5-OE cells under NNP-1 background. (**J**) Protein levels of Runx2 in ALKBH5-OE cells under NNP-1 background. (**K**) m^6^A methylation of *Runx2* mRNA in ALKBH5-OE cells under NNP-1 background. **(L** and **M)** Effects of ALKBH5 depletion and overexpression on the stability of *Runx2* mRNA. Both ALKBH5-KD and ALKDH-OE cells, as well as their corresponding controls, were treated with actinomycin D (2 mg/mL) at indicated time points. The mRNA levels of *Runx2* were measured by RT-qPCR. (**L**) ALKBH5-KD cells; (**M**) ALKBH5-OE cells. *P < 0.05; **P < 0.01; ***P < 0.001. ns represents no significant difference

*Runx2* was the most significantly downregulated gene after knockdown of ALKBH5 in the microarray result (Fig. 2A and Table S8), suggesting that *Runx2* might be regulated by ALKBH5. We identified five GGACU motifs in the 3’-UTR of the *Runx2* gene (Fig. 7A). To examine m^6^A modifications, we performed an anti-m^6^A immunoprecipitation assay and observed a significant decrease in m^6^A-modified *Runx2* 3’-UTR in degenerative IVDs compared to normal IVDs (Fig. 7B). The findings, coupled with those presented in Figure S11, indicate an inverse relationship between the m^6^A-modified Runx2 level and its corresponding mRNA and protein levels. Subsequently, we assessed the expression levels of Runx2 mRNA and protein in LPS-treated NNP1/2 cells. The data revealed that both mRNA and protein levels of Runx2 progressively increased in correlation with rising doses of LPS (Fig. 7C and D), suggesting a dose-dependent upregulation of Runx2 in response to LPS exposure. The in vitro experiments demonstrated a dose-dependent reduction in m^6^A-modified *Runx2* with increasing LPS concentrations (Fig. 7E).

To elucidate the interplay between ALKBH5 and Runx2, we examined the effects of ALKBH5 knockdown and overexpression on both Runx2 expression and m^6^A modification levels. ALKBH5 depletion resulted in reduced Runx2 mRNA and protein levels, accompanied by an increase in m^6^A-modified Runx2 in LENP-1 cells (Fig. 7F and H). Conversely, ALKBH5 overexpression correlated with elevated Runx2 mRNA and protein levels and a decrease in m^6^A-modified Runx2 in NNP-1 cells (Fig. 7I and K). These findings support the hypothesis that ALKBH5-mediated m^6^A demethylation may facilitate the stabilization of *Runx2* mRNA. RNA stability assays corroborated this, showing that ALKBH5 depletion significantly compromised *Runx2* mRNA stability in LENP-1 cells (Fig. 7L), while its overexpression enhanced *Runx2* mRNA stability in NNP-1 cells (Fig. 7M).

### Deficiency of YTHDF1 was required for the stability of *Runx2*mRNA

**Fig. 8:**
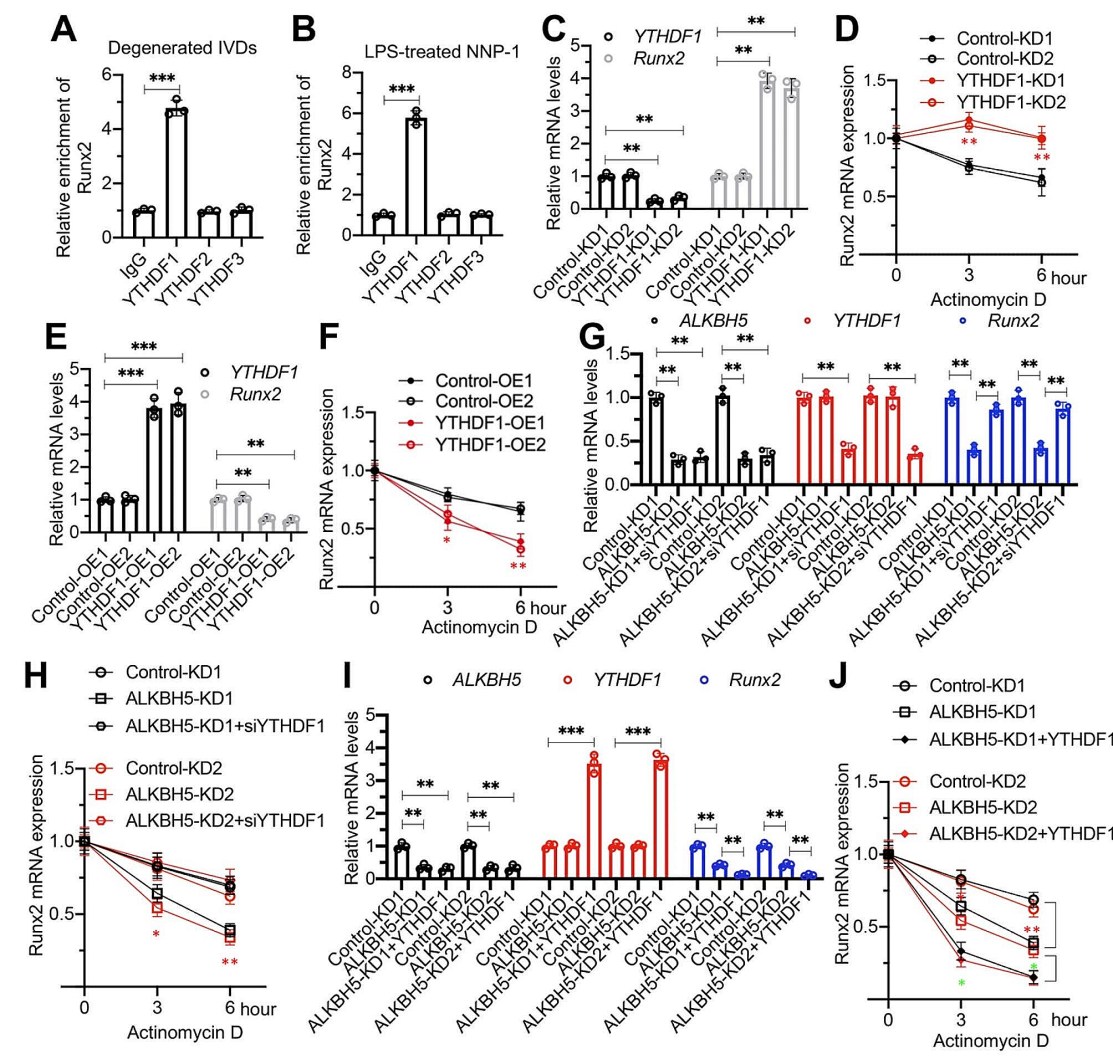
*Runx2* mRNA stability was dependent on YTHDF1 **(A** and **B)** Interaction between YTHDF1 and *Runx2* mRNA. RIP assays were performed using anti-YTHDF1, anti-YTHDF2, anti-YTHDF3, and IgG in three independent IVDs from LPS-administered mice (**A**) and LPS-treated NNP-1 cells (**B**). (**C**)*Runx2* mRNA levels in YTHDF1-KD cells. (**D**) Impact of YTHDF1 depletion on *Runx2* mRNA stability. The same cells as in (C) were treated with actinomycin D (2 mg/mL) at specified time points. The mRNA levels of *Runx2* were determined by RT-qPCR analysis. (**E**)*Runx2* mRNA levels in YTHDF1-OE cells. (**F**) Impact of YTHDF1 overexpression on *Runx2* mRNA stability. The same cells as in (E) were treated with actinomycin D (2 mg/mL) at specified time points. The mRNA levels of *Runx2* were determined by RT-qPCR analysis. (**G**) Influence of double knockdown of ALKBH5 and YTHDF1 on *Runx2* mRNA expression. (**H**) Impact of double knockdown of ALKBH5 and YTHDF1 on *Runx2* mRNA stability. The same cells as in (E) were treated with actinomycin D (2 mg/mL) at specified time points. The mRNA levels of *Runx2* were determined by RT-qPCR analysis. (**I**) Effect of overexpression of YTHDF1 in ALKBH5-KD cells on *Runx2* mRNA expression. (**J**) Effect of overexpression of YTHDF1 in ALKBH5-KD cells on *Runx2* mRNA stability. The same cells as in (I) were treated with actinomycin D (2 mg/mL) at specified time points. The mRNA levels of *Runx2* were determined by RT-qPCR analysis. *P < 0.05; **P < 0.01; ***P < 0.001

The YTHDF family proteins have been previously documented to control m^6^A-mediated mRNA destabilization [28, 32]. We have identified that YTHDF1 but not YTHDF2/3 was decreased in both degenerative IVDs and LPS-treated NNP cells. We next aimed to investigate whether YTHDF1 played a role in the ALKBH5-mediated destabilization of *Runx2* mRNA. Initially, we conducted a RIP assay followed by RT-qPCR analyses, which revealed a strong binding of YTHDF1 with *Runx2* mRNA in degenerative IVDs and LPS-treated NNP-1 cells (Fig. 8A and B). Notably, YTHDF2/3 did not exhibit significant binding to *Runx2* mRNA (Fig. 8A and B). Furthermore, upon specific knockdown of YTHDF1, we observed an increase in the expression of *Runx2* mRNA along with enhanced stability (Fig. 8C and D). Conversely, ectopic expression of YTHDF1 resulted in a significant decrease in both the expression and stability of *Runx2* mRNA in NNP-1 cells (Fig. 8E and F).

To ascertain the importance of YTHDF1 in the expression and stability of *Runx2*, we conducted rescue assays by overexpressing or depleting YTHDF1 in ALKBH5-KD cells (LENP-1 background). Remarkably, the targeted silencing of YTHDF1 in ALKBH5-KD cells substantially mitigated the suppression of *Runx2* mRNA expression caused by ALKBH5 loss, effectively restoring *Runx2* mRNA stability to levels comparable to Control-KD cells (Fig. 8G and H). In contrast, the forced overexpression of YTHDF1 led to a pronounced reduction in *Runx2* mRNA levels relative to ALKBH5-KD cells (Fig. 8I). Correspondingly, RNA stability assays revealed that YTHDF1 upregulation in ALKBH5-KD cells significantly compromised *Runx2* mRNA stability beyond that observed in ALKBH5-KD cells (Fig. 8J). Collectively, these results strongly suggest that ALKBH5-dependent m^6^A demethylation preserves the stability and expression of *Runx2* mRNA by counteracting YTHDF1-dependent mRNA destabilization.

### Knockout of ALKBH5 or overexpression of YTHDF1 in mice failed to induce IDD

**Fig. 9:**
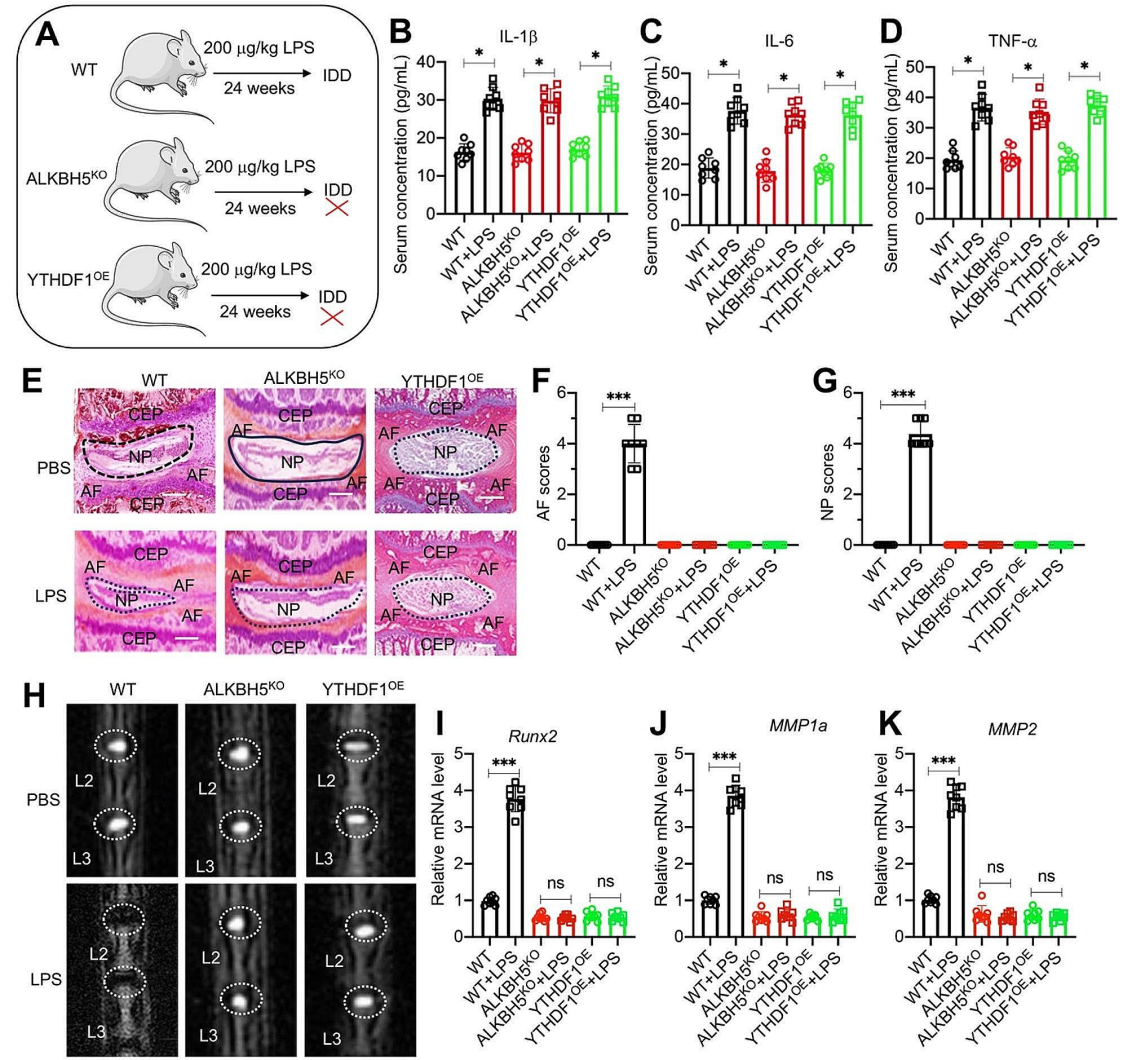
Chronic inflammation could not induce IDD in ALKBH5 ^KD^ and YTHDF1 ^OE^ mice (**A**) Experimental design depicting the study methodology. **(B-D)** Circulating concentrations of IL-1β (**B**), IL-6 (**C**), and TNF-α (**D**) in three groups of mice (WT, ALKBH5^KO^, and YTHDF1^OE^) injected with either PBS or LPS (*n* = 8 for each group). (**E**) Representative histological images of lumbar intervertebral discs (IVDs). Safranin O and fast green staining of L1/L2 lumbar IVDs from three groups of mice (WT, ALKBH5^KO^, and YTHDF1^OE^) injected with PBS or LPS. The positions of NP, AF, and CEP were labeled. Scale bars = 200 μm. **(F** and **G)** Quantified AF and NP scores in histological images of lumbar IVDs as depicted in (E). (**H**) Representative micro-CT images of lumbar IVDs (L2/L3) from three groups of mice (WT, ALKBH5^KO^, and YTHDF1^OE^) injected with PBS or LPS. **(I-K)** The mRNA expression levels of *Runx2*(**I**), *MMP1a*(**J**), and *MMP2*(**K**), in IVDs from three groups of mice (WT, ALKBH5^KO^, and YTHDF1^OE^) injected with PBS or LPS (*n* = 8 for each group). *P < 0.05; ***P < 0.001. ns represents no significant difference

Since both ALKBH5 and YTHDF1 were required for the expression of *Runx2*, we next aimed to evaluate the effects of ALKBH5 depletion and YTHDF1 overexpression on the incidence of IDD. Accordingly, we injected WT, ALKBH5 knockout (ALKBH^KO^), and YTHDF1 overexpression (YTHDF1^OE^) mice (*n* = 8 for each group) with 20 µg/kg LPS weekly (Fig. 9A). Following a 24-week administration of a low dose of LPS, we observed a comparable increase in serum levels of IL-1β, IL-6, and TNF-α across all groups of mice (Fig. 9B and D). Notably, degenerative lumbar IVDs were only observed in WT mice, while ALKBH^KO^ and YTHDF1^OE^ mice showed no signs of IDD following LPS administration (Fig. 9E and H). Furthermore, we evaluated the mRNA levels of *Runx2* and its downstream target genes in lumbar IVDs from these 6 groups of mice. Our RT-qPCR analysis revealed that the expression of *Runx2*, *MMP1a/2/3/9/10*, and *ADAMTS9/10/13* could not be induced by LPS and they remained consistently low in both ALKBH^KO^ and YTHDF1^OE^ mice after LPS administration (Fig. 9I and K and S14).

### The ALKBH5/Runx2/MMPs/ADAMTSs signaling was conserved in human IDD patients with chronic inflammation

**Fig. 10:**
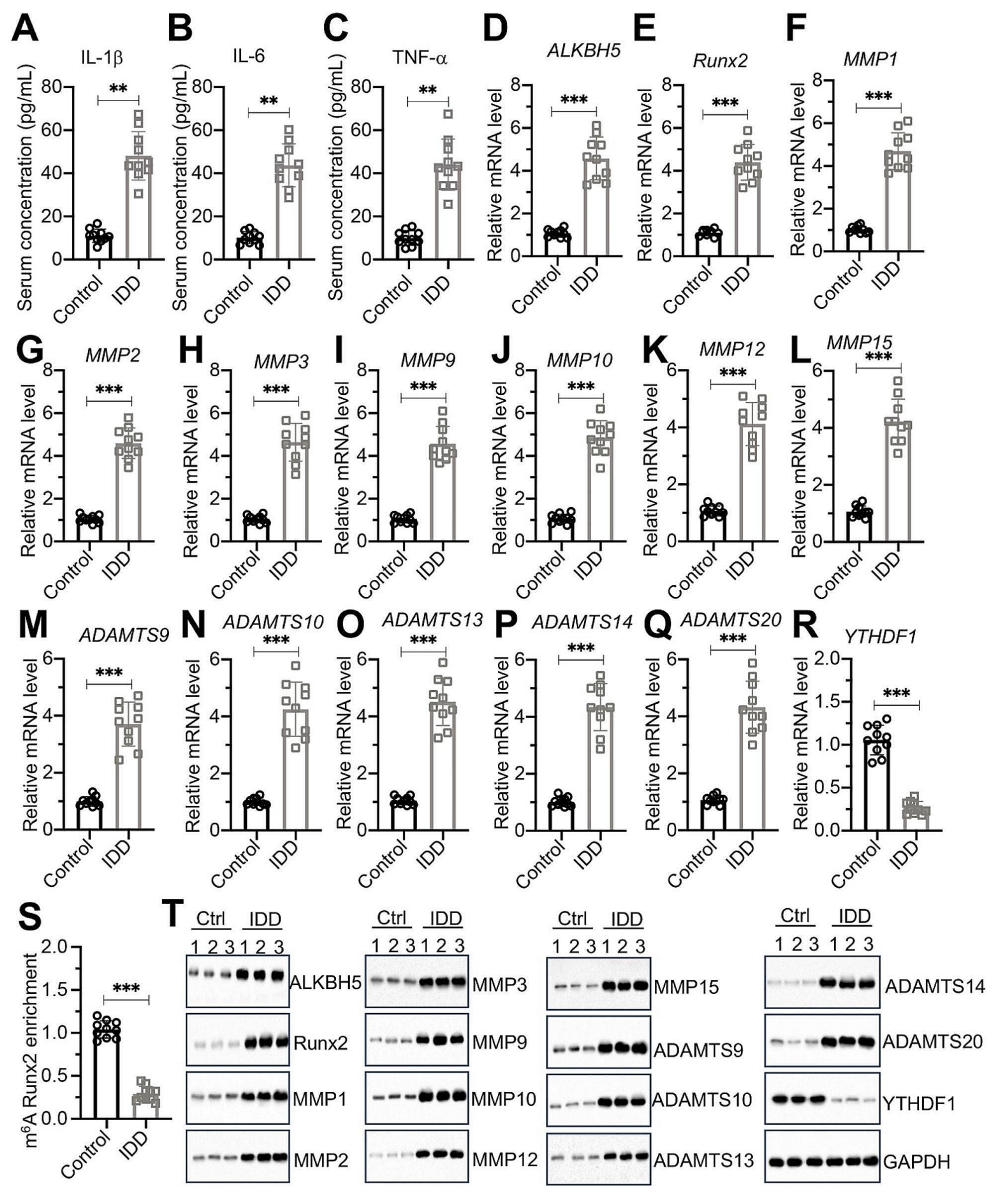
Expression levels of ALKBH5, Runx2, MMPs and ADAMTSs were increased in human IVD biopsies with chronic inflammation **(A-C)** Circulating concentrations of IL-1β (**A**), IL-6 (**B**), and TNF-α (**C**) in Control and IDD patients (*n* = 10 for each group). **(D-S)** The mRNA levels of *ALKBH5*(**D**), *Runx2*(**E**), *MMP1*(**F**), *MMP2*(**G**), *MMP3*(**H**), *MMP9*(**I**), *MMP10*(**J**), *MMP12*(**K**), *MMP15*(**L**), *ADAMTS9*(**M**), *ADAMTS10*(**N**), *ADAMTS13*(**O**), *ADAMTS14*(**P**), *ADAMTS20*(**Q**), and *YTHDF1*(**R**), in IVDs from Control and IDD patients (*n* = 10 for each group). (**S**) m^6^A methylation of *Runx2* mRNA in IVDs from Control and IDD patients (*n* = 10 for each group). (T) Protein levels of ALKBH5, Runx2, MMP1, MMP2, MMP3, MMP9, MMP10, MMP12, MMP15, ADAMTS9, ADAMTS10, ADAMTS13, ADAMTS14, ADAMTS20, and YTHDF1 in IVDs from Control and IDD patients (*n* = 3 for each group)

Our experimental results in mouse NP cells and IVD tissues indicate that the ALKBH5/Runx2/MMPs/ADAMTSs signaling pathway plays a crucial role in responding to chronic inflammation and regulating IDD occurrence. To confirm the conservation of this mechanism, we collected lumbar disc samples from ten donors who succumbed to accidents and presented no evidence of inflammation and IDD. Additionally, we obtained samples from ten patients exhibiting chronic inflammation who were undergoing disc replacement surgery as a treatment for IDD. Following the confirmation of elevated pro-inflammatory cytokines IL-1β, IL-6, and TNF-α in the IDD group compared to the control group via ELISA (Fig. 10A and C), we measured the expression of the ALKBH5/Runx2/MMPs/ADAMTSs signaling molecules in these IVD tissues. RT-qPCR analysis revealed that the expressions of *ALKBH5*, *Runx2*, *MMP1*, *MMP2*, *MMP3*, *MMP9*, *MMP10*, *MMP12*, *MMP15*, *ADAMTS9*, *ADAMTS10*, *ADAMTS13*, *ADAMTS14*, and *ADAMTS20* were significantly higher in IDD patients than in the control group, while YTHDF1 expression was lower (Fig. 10D and R). Moreover, we assessed the m^6^A modification levels of Runx2, finding significantly reduced m^6^A modifications of Runx2 in the IDD group compared to controls (Fig. 10S). Protein assays conducted on three selected IVD samples from both the control and IDD groups showed that the protein levels of ALKBH5, Runx2, and the aforementioned MMPs and ADAMTSs were significantly elevated in the IDD group, with YTHDF1 expression notably reduced (Fig. 10T). These findings suggest the presence of a conserved regulatory mechanism mediated by the ALKBH5/Runx2/MMPs/ADAMTSs signaling axis in patients with IDD under chronic inflammation.

## Discussion

**Fig. 11:**
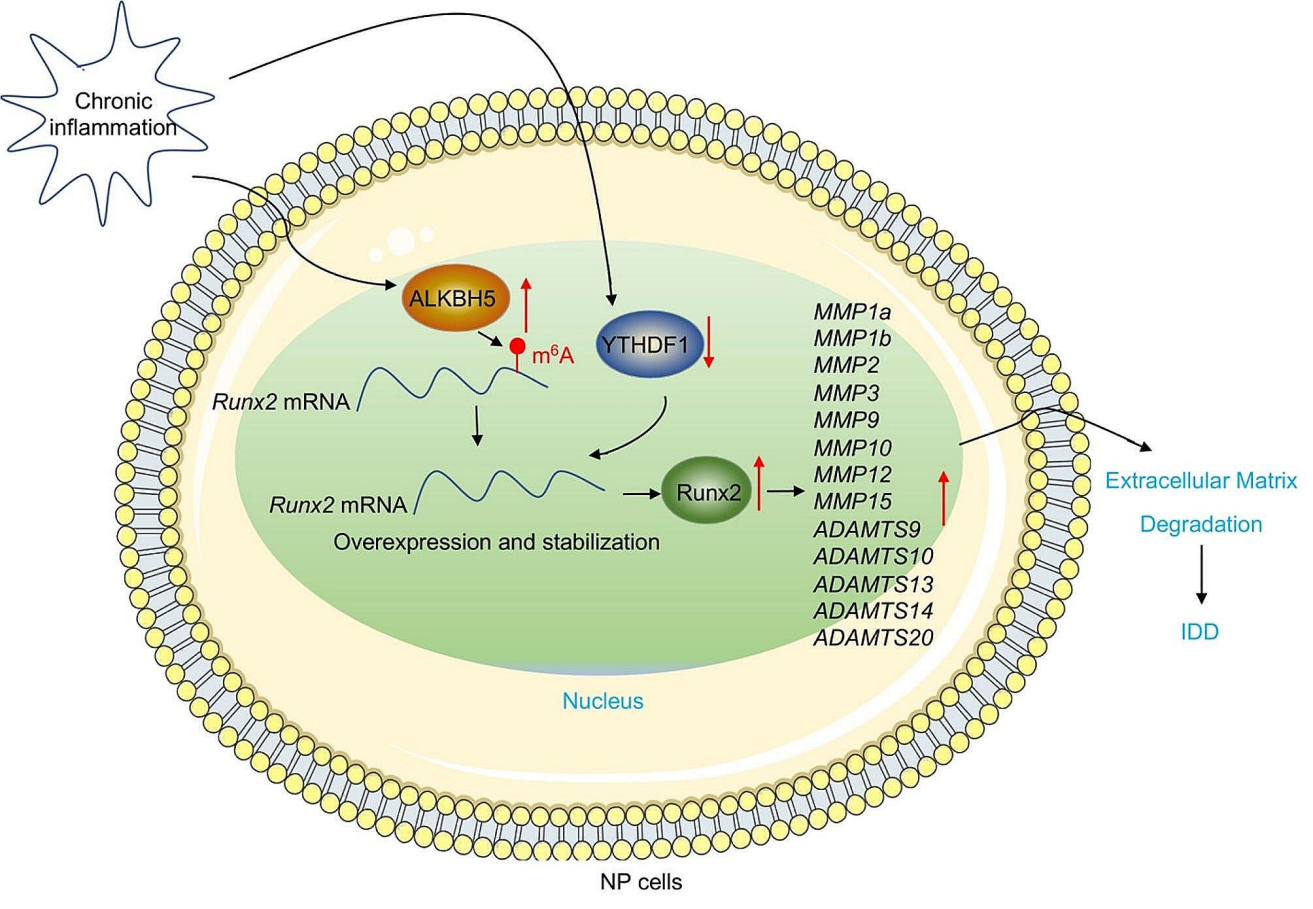
Proposed model illustrating the mechanism by which ALKBH5-mediated m^6^A demethylation of *Runx2* mRNA promotes IDD incidenceThe chronic inflammation microenvironment leads to the induction of ALKBH5 and a decrease in YTHDF1 within IVDs. Upregulation of ALKBH5 facilitates m^6^A demethylation, enhancing the stability and expression of *Runx2* mRNA in a YTHDF1-dependent manner. The increased expression of Runx2 serves as a master transcription factor, leading to the upregulation of multiple members of *MMP* and *ADAMTS* genes. This cascade results in ECM degradation and ultimately contributes to the incidence of IDD

This degenerative condition of IDD is commonly associated with aging, mechanical stress, genetic predisposition, and various environmental factors, including chronic inflammation [1, 2]. However, the precise molecular mechanisms underlying how chronic inflammation leads to IDD remain unclear. In this study, we present evidence supporting the upregulation of ALKBH5 and downregulation of YTHDF1 in the chronic inflammation microenvironment, resulting in increased expression and stability of *Runx2* mRNA. Runx2 serves as a master transcription factor, inducing the expression of *MMPs* and *ADAMTSs*, which promote ECM degradation in IVDs and contribute to IDD incidence (Fig. 11).

Eukaryotic gene expression is dynamically regulated by multiple factors, including transcription factors/transcriptional coactivators/corepressors [33], non-coding RNAs [34], and epigenetic modifications [35]. Among these factors, RNA m^6^A modification, the most prevalent RNA modification at the posttranscriptional level, has emerged as a novel and intriguing player in the pathogenesis of IDD, as in many other diseases [22–26]. For instance, altered expression and activity of m^6^A “writers” and “erasers” have been observed in degenerated discs, indicating disrupted m^6^A dynamics [36, 37]. Li et al. demonstrated that the upregulation of WTAP in senescent nucleus pulposus cells (NPCs) leads to m^6^A modification of NORAD (non-coding RNA activated by DNA damage), thereby promoting IDD through a YTHDF2-dependent mechanism [36]. NORAD deficiency reduces sequestration of PUM1/2 (Pumilio RNA Binding Family Member 1), suppressing the expression of *E2F3* (E2F transcription factor 3) mRNAs and promoting cellular senescence [36]. Additionally, elevated expression of ALKBH5 has been observed during the IDD process and in senescent NPCs [37]. Li et al. revealed that decreased KDM4A-mediated H3K9me3 modification leads to ALKBH5 overexpression [37]. Functionally, ALKBH5 induces NPC senescence by promoting the demethylation of *DNMT3B* (DNA methyltransferase 3 beta) mRNA via less YTHDF2 recognition [37]. Similarly, in our study, we also observed the accumulation of ALKBH5 in degenerated IVDs. The specific changes of ALKBH5 and YTHDF1 in degenerative IVDs from chronically inflamed mice and LPS-exposed NP cells suggest that these two proteins, rather than other m^6^A regulators, play a unique role in mediating *Runx2* expression and stability in response to inflammation. Importantly, the inflammation microenvironment fails to induce IDD in both ALKBH^KO^ and YTHDF1^OE^ mice, suggesting that ALKBH5 and YTHDF1 hold potential as therapeutic targets for treating IDD patients with chronic inflammation.

Runx2, a master regulator of skeletal development, is primarily recognized for its crucial involvement in osteoblast differentiation and bone formation [15–17]. However, emerging evidence indicates that Runx2 also plays a pivotal role in maintaining the homeostasis and deterioration of IVDs [18–21]. Upregulation of Runx2 expression has been observed in degenerative IVDs in comparison to healthy discs [17, 21]. This upregulation is believed to be a response to the microenvironmental changes that occur during disc degeneration. Runx2 has demonstrated its regulatory control over multiple enzymes, including MMPs and ADAMTSs, responsible for the degradation of ECM components within the disc [17–20]. Moreover, Runx2 has been implicated in modulating crucial signaling pathways involved in the pathogenesis of IDD [21]. Its interaction and regulation with key factors such as transforming growth factor-beta (TGF-β), bone morphogenetic proteins (BMPs), and Wnt/β-catenin signaling have been observed [1–3]. Disruption of these pathways has been associated with the loss of disc cellularity, aberrant remodeling of the matrix, and inflammation, all of which are critical aspects of IDD [1–3]. However, the specific mechanism underlying the upregulation of Runx2 in IDD pathogenesis remains unknown. In this study, we demonstrate for the first time that the dysregulation of an m^6^A demethylation mechanism, dependent on ALKBH5, is responsible for the overexpression of *Runx2* in degenerative discs. This novel finding uncovers the underlying mechanism of *Runx2* overexpression and provides a new avenue for investigating the role of m^6^A methylation in the pathogenesis of IDD.

The mouse genome encodes 23 *MMP* genes and 19 *ADAMTS* genes. Several of these genes, such as *MMP2*, *MMP9*, *MMP13*, *ADAMTS4*, and *ADAMTS5*, have previously been reported to be overexpressed in degenerative discs [18, 19, 31]. In our study, we have identified 8 *MMPs* and 5 *ADAMTSs* whose expression levels were found to be upregulated following LPS challenge. Remarkably, all 13 of these genes are regulated by Runx2. These novel findings significantly contribute to our understanding of how Runx2 governs the regulation of target genes during the pathogenesis of IDD. In this study, we administered recombinant Runx2 protein, MMP1a, and ADAMTS10 individually, as well as MMP1a + ADAMTS10 combination, near the lumbar discs of mice. Our findings revealed complete degradation of lumbar IVDs in mice injected with Runx2, while the efficacy of injections with MMP1a, ADAMTS10, and MMP1a + ADAMTS10 was notably inferior to that of Runx2 alone. This directly demonstrates that overexpression of Runx2 in IVD cells directly leads to IDD, underscoring the involvement of numerous MMPs and ADAMTSs in the degenerative process of IDD. Injection of individual MMPs or ADAMTSs, or their combinations, failed to replicate the efficacy achieved by Runx2 alone. Moreover, we observed elevated expression levels of ALKBH5, Runx2, MMPs, and ADAMTSs, alongside reduced expression of YTHDF1, in human IDD samples characterized by chronic inflammation. These findings parallel those observed in mouse models exhibiting chronic inflammation, suggesting the existence of a conserved regulatory pathway involving ALKBH5 and YTHDF1-mediated m^6^A modification in both humans and mice. This pathway modulates Runx2 and its downstream signaling pathways, playing a crucial regulatory role in the onset of IDD.

In summary, our study highlights m^6^A modification as a novel mechanism underlying IDD in the context of chronic inflammation. The upregulation of ALKBH5 promotes m^6^A demethylation, leading to increased stability and expression of *Runx2* mRNA, thereby contributing to ECM degradation within the discs. Our data strongly support the concept that targeting the ALKBH5/Runx2 axis holds great promise as a strategy for preventing IDD incidence.

## Electronic supplementary material

Below is the link to the electronic supplementary material.

Supplementary Material 1

Supplementary Material 2

## Data Availability

The datasets used and/or analyzed during the present study are available from the corresponding author on reasonable request.
