# Correction: ALKBH5-mediated m^6^A demethylation of *Runx2* mRNA promotes extracellular matrix degradation and intervertebral disc degeneration

**DOI:** 10.1186/s13578-024-01330-5

**Published:** 2024-12-21

**Authors:** Yu Lei, Enyu Zhan, Chao Chen, Yaoquan Hu, Zhengpin Lv, Qicong He, Xuenan Wang, Xingguo Li, Fan Zhang

**Affiliations:** Department of Orthopedics, The First Affiliated Hospital of Kunming Medical University, 295 Xichang Rd, Wuhua District, Kunming, 650032 Yunnan China

**Correction: Cell & Bioscience (2024) 14:79** 10.1186/s13578-024-01264-y

In this article [1], the author would like to correct the product information of ADAMTS10 in the section of "**Administration of recombinant Runx2, MMP1a, and ADAMTS proteins near lumbar discs**".

Incorrect text:

ADAMTS10 protein (R&D Systems, Inc., Shanghai, China; #946-AD-020; 20 mg/kg)

Correct text:

ADAMTS10 (CUSABIO; Wuhan, Hubei, China; #CSB-EP001299MO; 20 mg/kg).
