# Supplementary material for: ALKBH5-mediated m6A demethylation of Runx2 mRNA promotes extracellular matrix degradation and intervertebral disc degeneration
Source: Cell Biosci. 2024 Jun 14;14:79. doi: 10.1186/s13578-024-01264-y (PMC11179301; doi:10.1186/s13578-024-01264-y)
Supplement: Supplementary file 1 — Supplementary Material 1 [file 13578_2024_1264_MOESM1_ESM.docx]

**
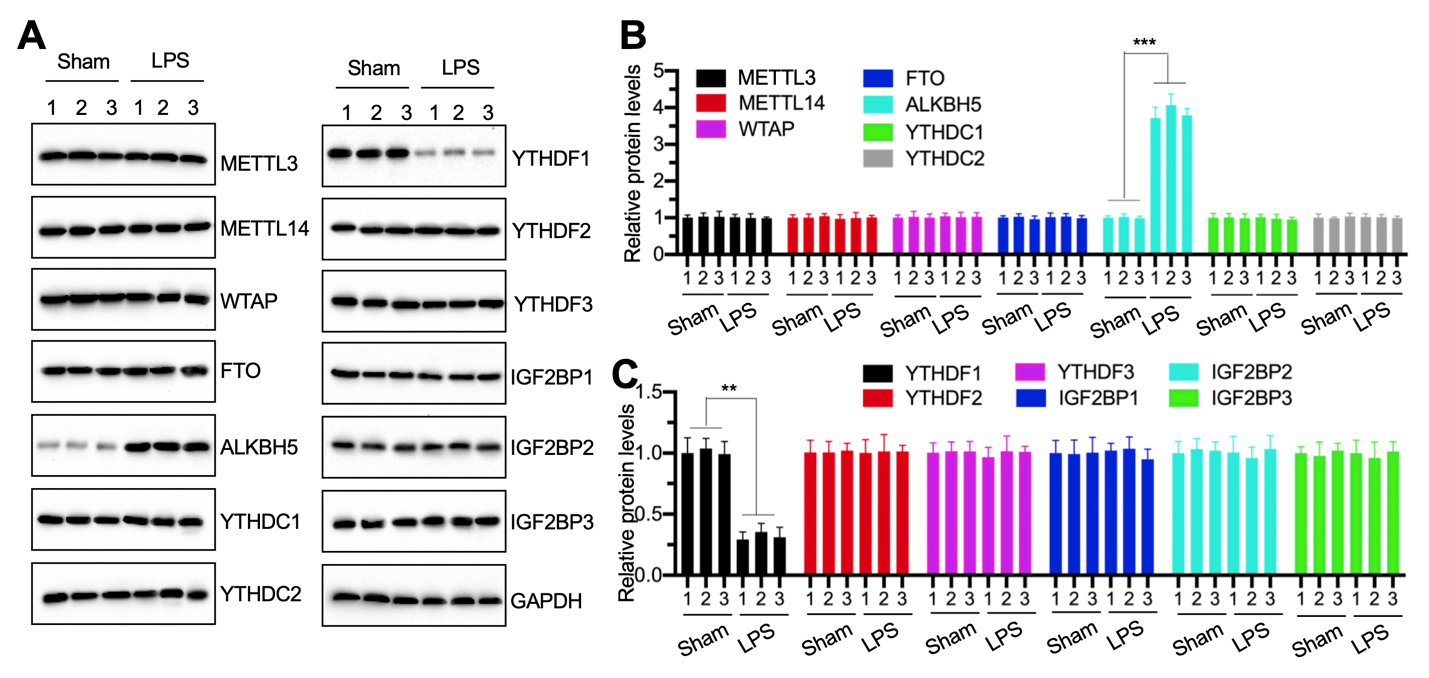
Figure S1. The protein levels of** **m^6^A methyltransferases, demethylases, and m^6^A modification binding proteins in IVDs from sham and LPS-challenged mice**

**(A)** Western blotting results. Equal protein extracts (50 µg) of three representative IVDs from sham and LPS groups were loaded to examine protein levels of METTL3, METTL14, WTAP, FTO, ALKBH5, YTHDC1, YTHDC2, YTHDF1, YTHDF2, YTHDF3, IGF2BP1, IGF2BP2, IGF2BP3, and GAPDH (loading control). **(B and C)** Quantified protein levels. Protein signals in (A) were quantified using Image J software and then normalized to their corresponding loading controls. ***P* < 0.01; ****P* < 0.001.

**
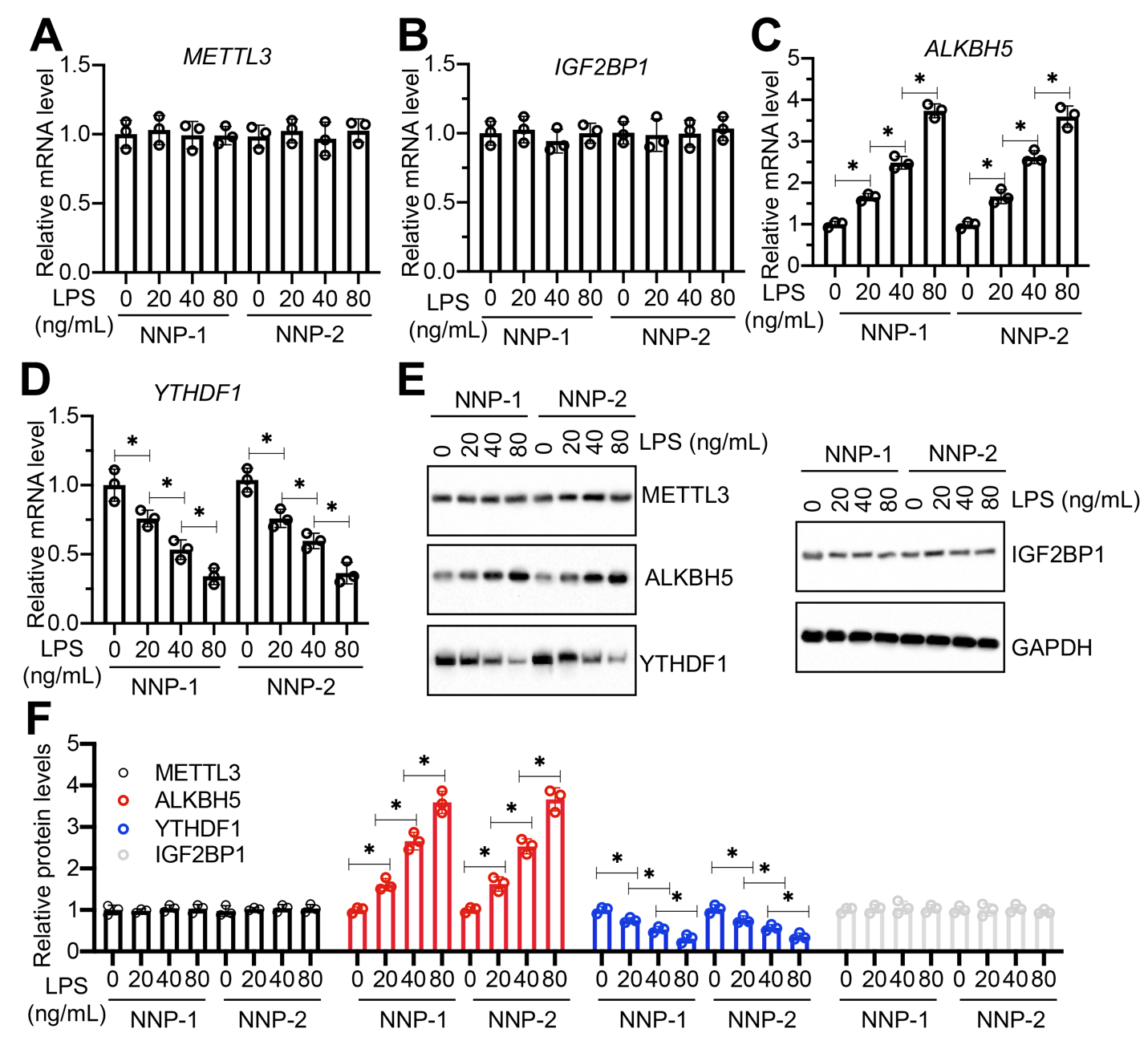
**

**Figure S2. Altered expression of ALKBH5 and YTHDF1 was observed in LPS-treated NNP cells**

**(A-D)** mRNA levels of *METTL3/ALKBH5/YTHDF1/IGF2BP1* in LPS treated NNP cells. Two primary NNP cell lines (#1 and #2) were treated with varying doses of LPS (0, 20, 40, and 80 ng/mL) at 37°C for 6 h. RNA samples from LPS-treated cells were subjected to RT-qPCR analyses to measure mRNA levels of *METTL3* **(A)**, *IGF2BP1* **(B)**, *ALKBH5* **(C)**, and *YTHDF1* **(D)**. **(E and F)** Protein levels of METTL3/ALKBH5/YTHDF1/IGF2BP1 in LPS treated NNP cells. The same cells as used in (A) were subjected to total protein extraction, followed by immunoblots to examine protein levels of METTL3, ALKBH5, YTHDF1, IGF2BP1, and GAPDH. **(E)** Western blotting results. **(F)** Quantified protein levels. **P* < 0.05.

**
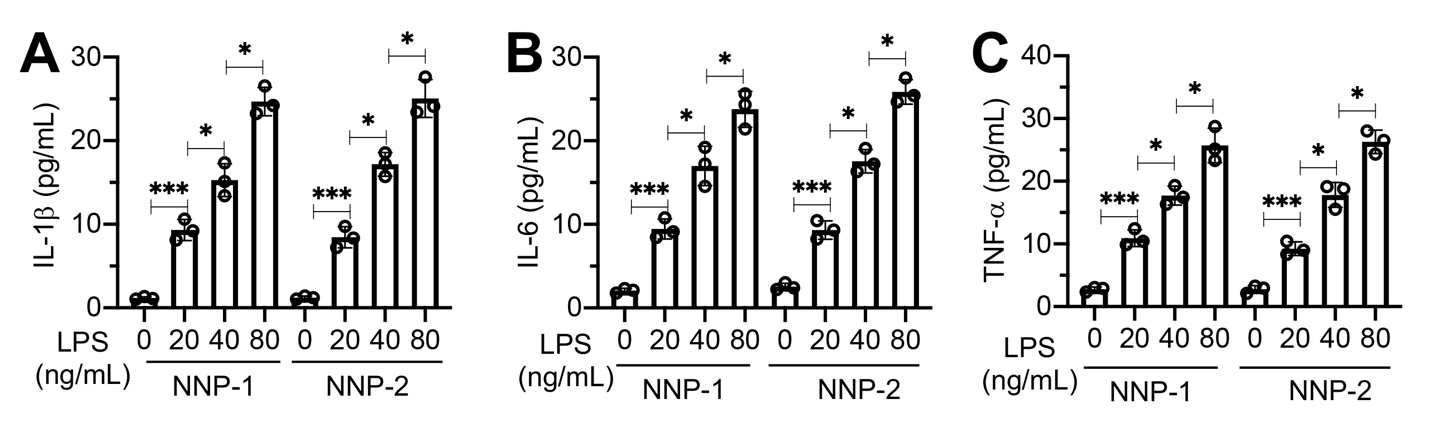
**

**Figure S3. Levels of proinflammatory cytokines in the supernatants of cell cultures treated with LPS**

Two primary NNP cell lines (#1 and #2) were treated with increasing doses of LPS (0, 20, 40, and 80 ng/mL) at 37°C for 6 hours. Following treatment, the supernatants from the cell cultures were collected for ELISA analysis to measure the levels of proinflammatory cytokines, specifically IL-1β **(A)**, IL-6 **(B)**, and TNF-α **(C)**. **P* < 0.05; ****P* < 0.001.

**
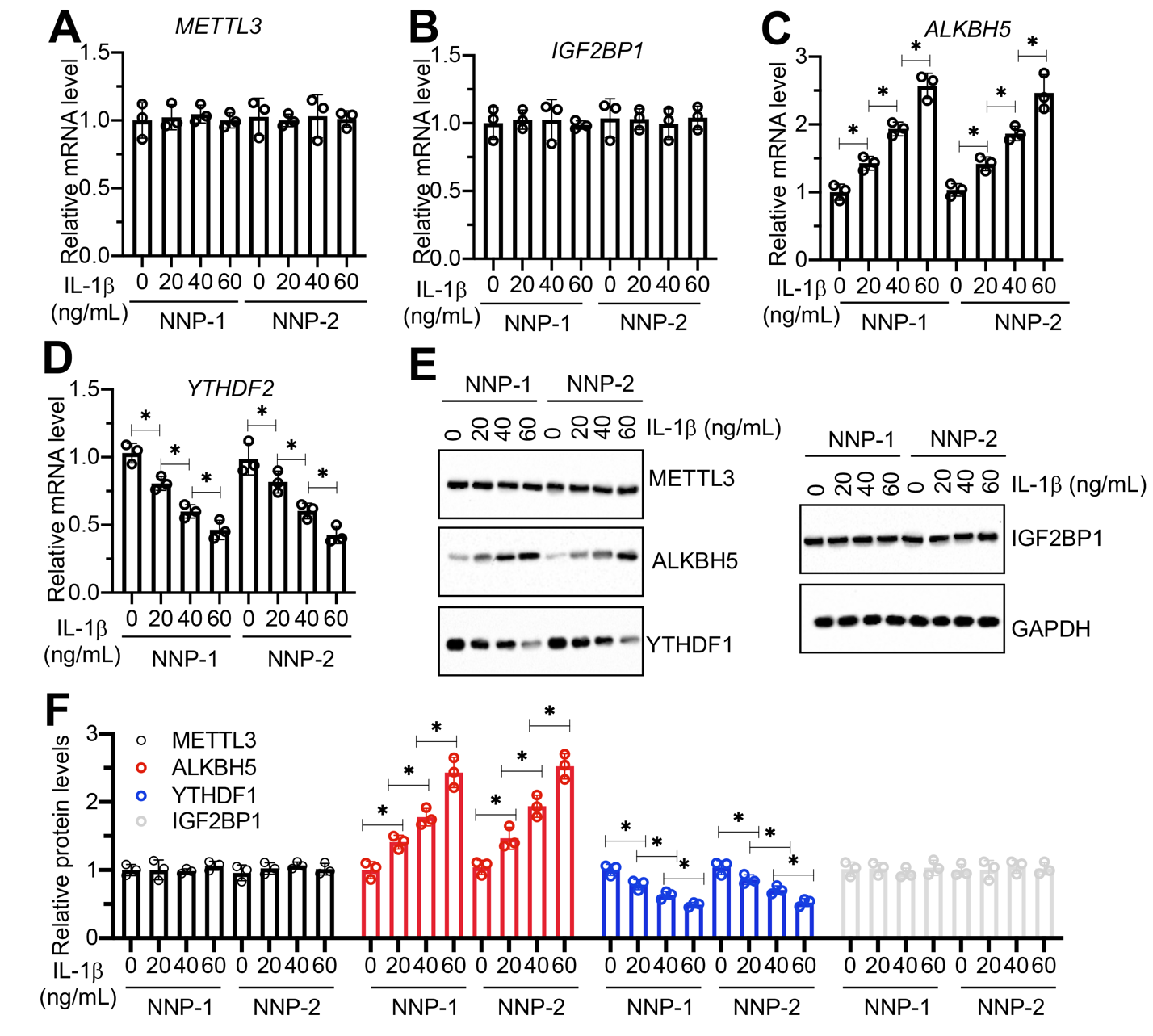
**

**Figure S4. Altered expression of ALKBH5 and YTHDF1 was observed in IL-1β-treated NNP cells**

**(A-D)** mRNA levels of *METTL3/ALKBH5/YTHDF1/IGF2BP1* in IL-1β treated NNP cells. Two primary NNP cell lines (#1 and #2) were treated with varying doses of IL-1β (0, 20, 40, and 60 ng/mL) at 37°C for 6 h. RNA samples from IL-1β-treated cells were subjected to RT-qPCR analyses to measure mRNA levels of *METTL3* **(A)**, *IGF2BP1* **(B)**, *ALKBH5* **(C)**, and *YTHDF1* **(D)**. **(E and F)** Protein levels of METTL3/ALKBH5/YTHDF1/IGF2BP1 in IL-1β treated NNP cells. The same cells as used in (A) were subjected to total protein extraction, followed by immunoblots to examine protein levels of METTL3, ALKBH5, YTHDF1, IGF2BP1, and GAPDH. **(E)** Western blotting results. **(F)** Quantified protein levels. **P* < 0.05.


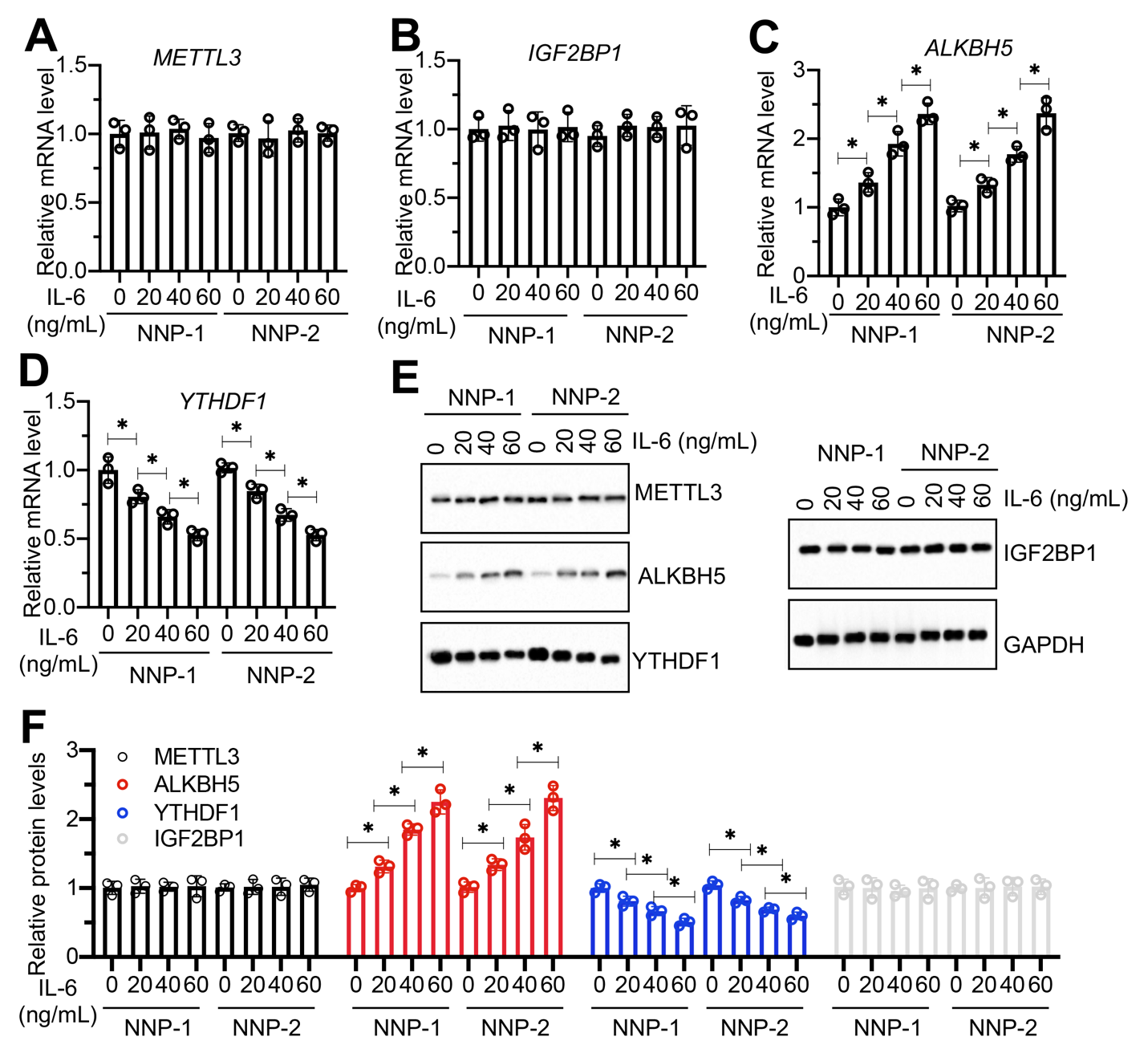


**Figure S5. Altered expression of ALKBH5 and YTHDF1 was observed in IL-6-treated NNP cells**

**(A-D)** mRNA levels of *METTL3/ALKBH5/YTHDF1/IGF2BP1* in IL-6 treated NNP cells. Two primary NNP cell lines (#1 and #2) were treated with varying doses of IL-6 (0, 20, 40, and 60 ng/mL) at 37°C for 6 h. RNA samples from IL-6-treated cells were subjected to RT-qPCR analyses to measure mRNA levels of *METTL3* **(A)**, *IGF2BP1* **(B)**, *ALKBH5* **(C)**, and *YTHDF1* **(D)**. **(E and F)** Protein levels of METTL3/ALKBH5/YTHDF1/IGF2BP1 in IL-6 treated NNP cells. The same cells as used in (A) were subjected to total protein extraction, followed by immunoblots to examine protein levels of METTL3, ALKBH5, YTHDF1, IGF2BP1, and GAPDH. **(E)** Western blotting results. **(F)** Quantified protein levels. **P* < 0.05.

**
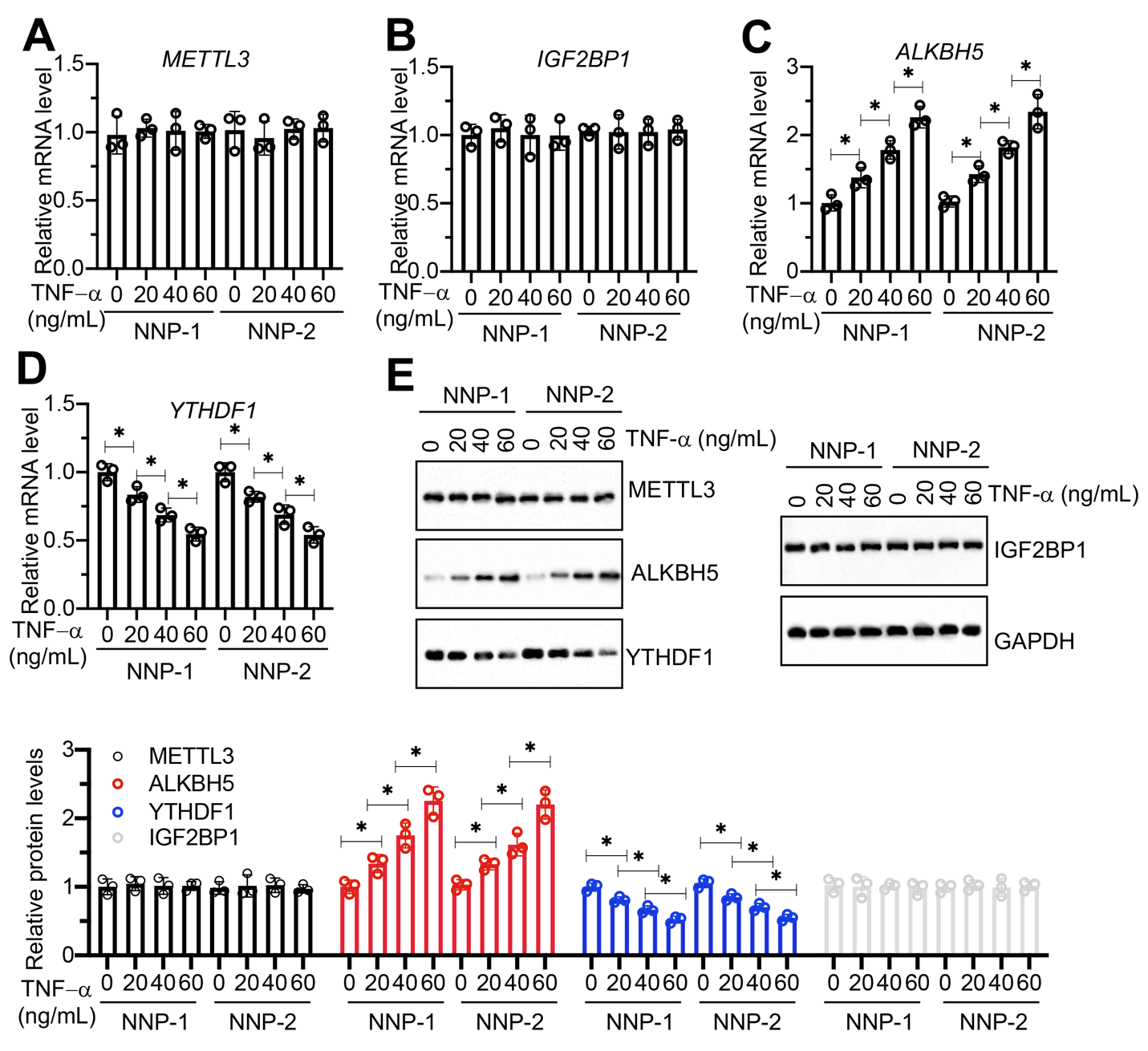
Figure S6. Altered expression of ALKBH5 and YTHDF1 was observed in TNF-α-treated NNP cells**

**(A-D)** mRNA levels of *METTL3/ALKBH5/YTHDF1/IGF2BP1* in TNF-α treated NNP cells. Two primary NNP cell lines (#1 and #2) were treated with varying doses of TNF-α (0, 20, 40, and 60 ng/mL) at 37°C for 6 h. RNA samples from TNF-α-treated cells were subjected to RT-qPCR analyses to measure mRNA levels of *METTL3* **(A)**, *IGF2BP1* **(B)**, *ALKBH5* **(C)**, and *YTHDF1* **(D)**. **(E and F)** Protein levels of METTL3/ALKBH5/YTHDF1/IGF2BP1 in TNF-α treated NNP cells. The same cells as used in (A) were subjected to total protein extraction, followed by immunoblots to examine protein levels of METTL3, ALKBH5, YTHDF1, IGF2BP1, and GAPDH. **(E)** Western blotting results. **(F)** Quantified protein levels. **P* < 0.05.


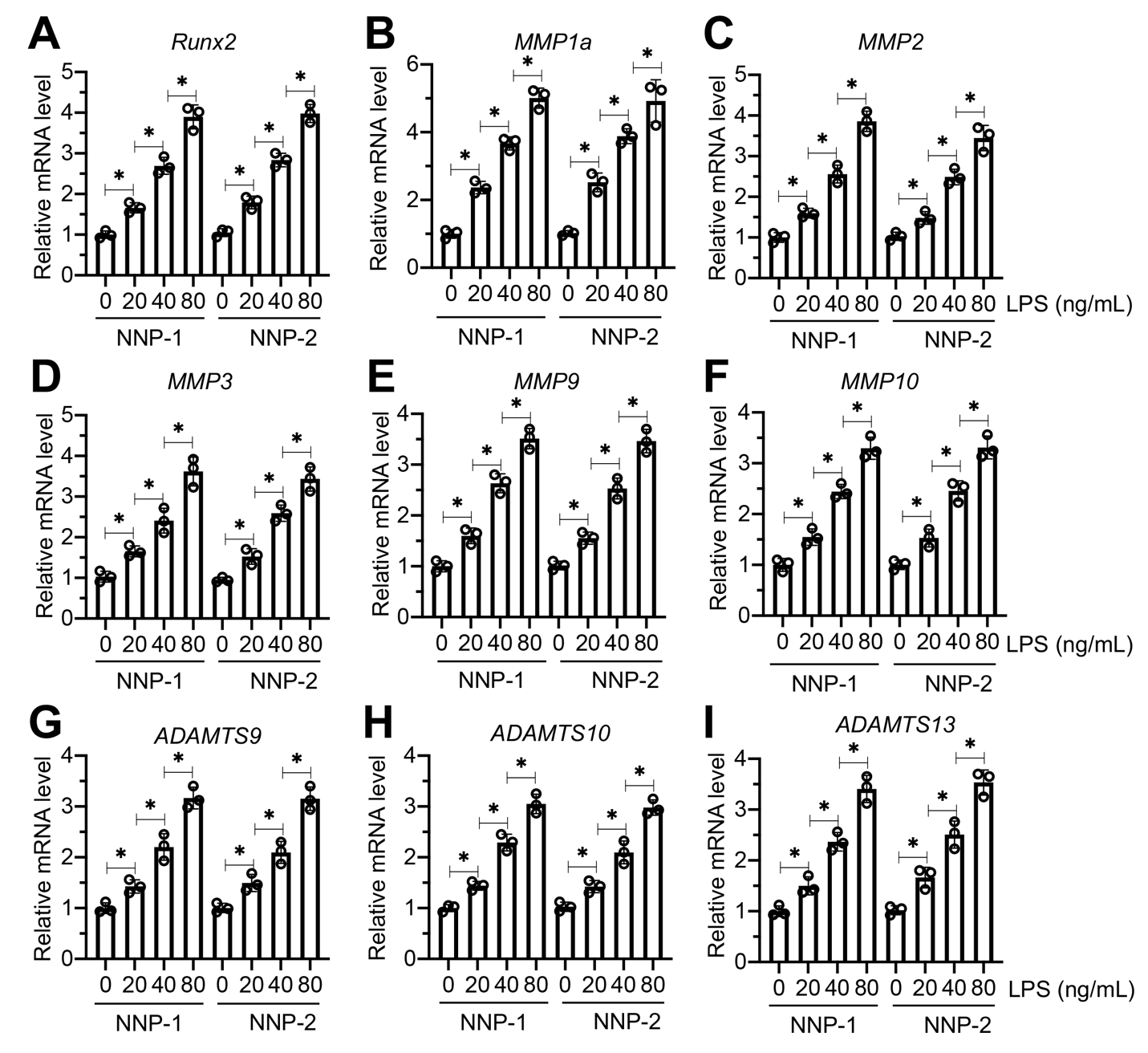


**Figure S7. mRNA levels of *Runx2/MMP1a/MMP2/MMP3/MMP9/MMP10* and *ADAMTS9/10/13* in LPS-treated NNP cells**

Two primary NNP cell lines (#1 and #2) were treated with varying doses of LPS (0, 20, 40, and 80 ng/mL) at 37°C for 6 h. RNA samples from LPS-treated cells were subjected to RT-qPCR analyses to measure mRNA levels of *Runx2* **(A)**, *MMP1a* **(B)**, *MMP2* **(C)**, *MMP3* **(D)**, *MMP9* **(E)**, *MMP10* **(F)**, *ADAMTS9* **(G)**, *ADAMTS10* **(H)**, and *ADAMTS13* **(I)**. **P* < 0.05.

**
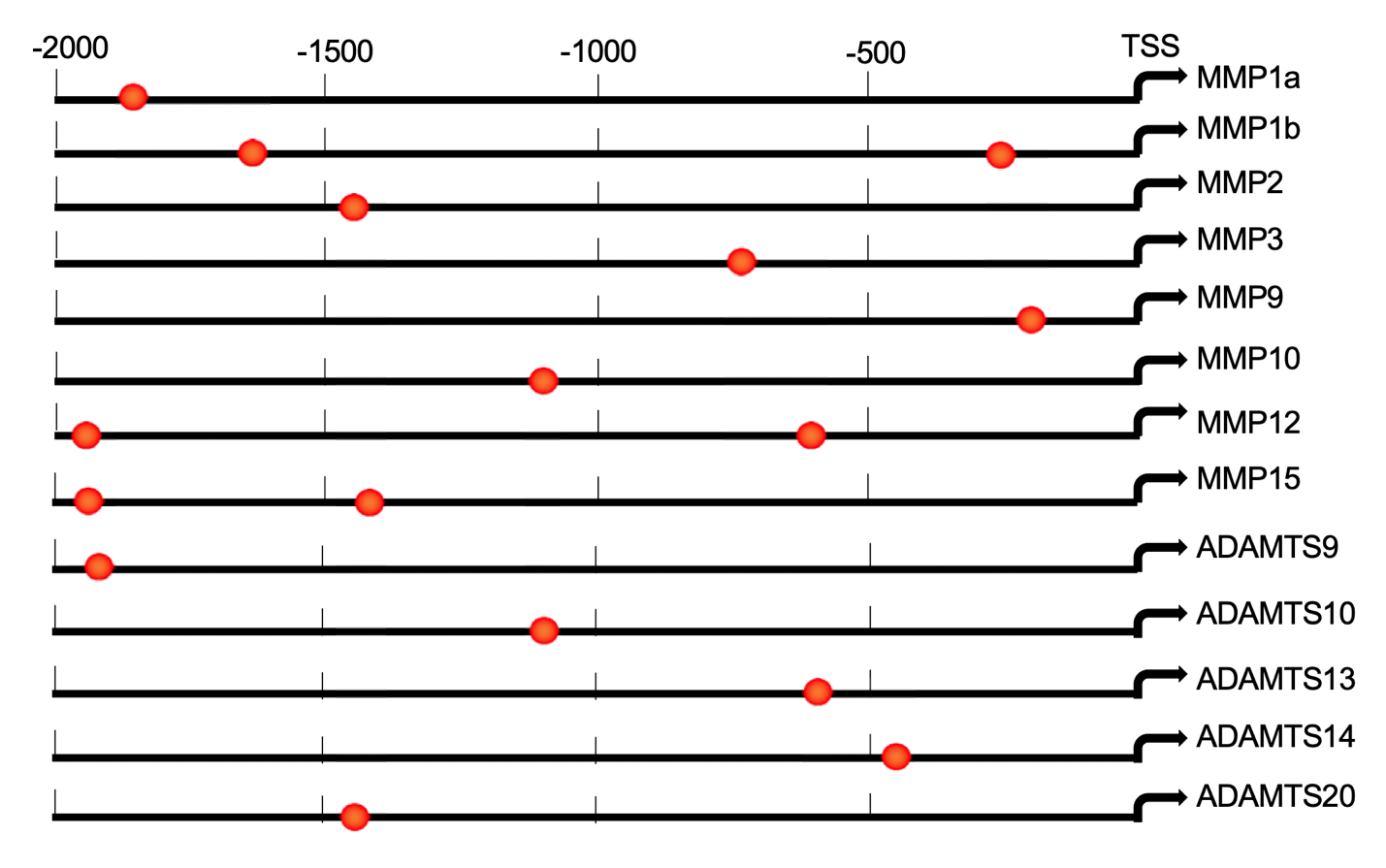
**

**Figure S8. Presence of the Runx2 binding site in the promoters of *MMP* and *ADAMTS* genes dependent on ALKBH5**

The 2000 bp-long promoters of 13 genes, including *MMP1a*, *MMP1b*, *MMP2*, *MMP3*, *MMP9*, *MMP10*, *MMP12*, *MMP15*, *ADAMTS9*, *ADAMTS10*, *ADAMTS13*, *ADAMTS14* and *ADAMTS20*, were analyzed to identify the binding site of Runx2 using its consensus sequence TGTGGT. The binding sites of Runx2 are indicated by red circles.


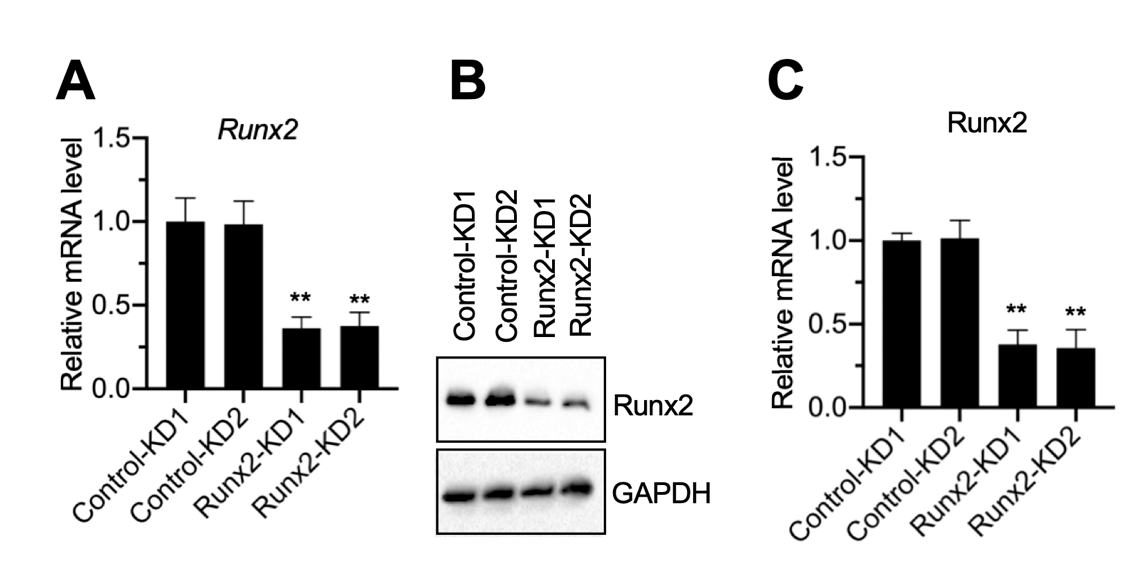


**Figure S9. Runx2 mRNA and protein levels in Runx2-knockdown (KD) cells**

LENP-1 cells were transfected with lentiviral vectors expressing nontargeting pLKO.1 control shRNA or Runx2-specific targeting shRNAs to generate Control-KD (#1 and #2) and Runx2-KD (#1 and #2) cell lines. RNA isolation and protein extraction were performed on these stable cell lines to assess Runx2 mRNA and protein levels, respectively. **(A)** *Runx2* mRNA levels were measured by qRT-PCR. **(B)** Western blotting results showing Runx2 protein levels. **(C)** Quantification of Runx2 protein levels. ***P* < 0.01.

**
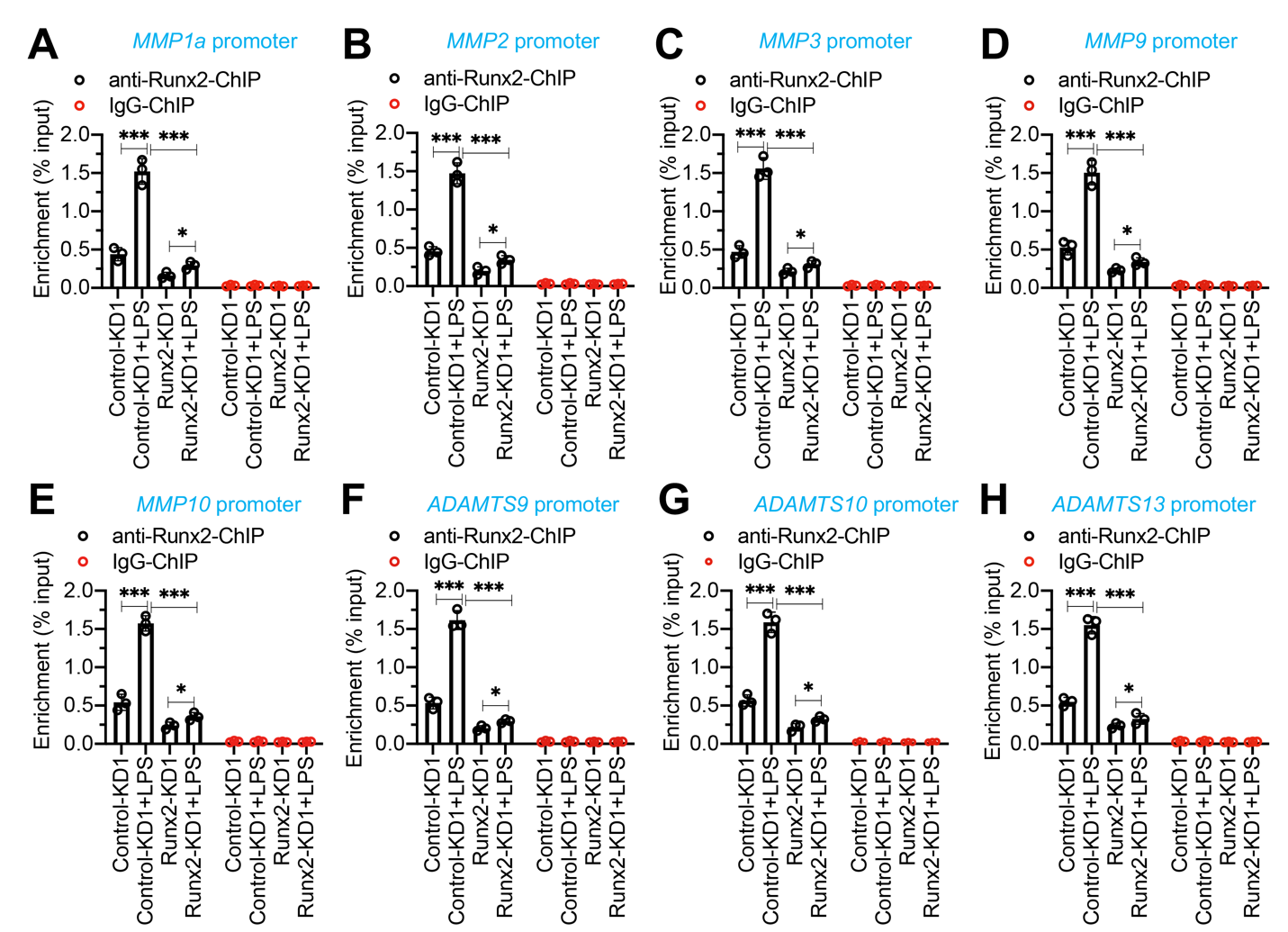
**

**Figure S10. The binding of Runx2 on the promoters of *MMP1a/2/3/9/10* and *ADAMTS9/10/13* was significantly decreased in Runx2-KD cells**

The Control-KD1 and Runx2-KD1 cells were treated with or without 80 ng/mL LPS for 6 hr, followed by ChIP assay with anti-Runx2- or IgG-coated protein G agarose. Input and output DNA samples were subjected to RT-qPCR analyses to measure the enrichment of Runx2 on the promoters of *MMP1a* **(A)**, *MMP2* **(B)**, *MMP3* **(C)**, *MMP9* **(D)**, *MMP10* **(E)**, *ADAMTS9* **(F)**, *ADAMTS10* **(G)**, and *ADAMTS13* **(H)**. **P* < 0.05; ****P* < 0.001.

**
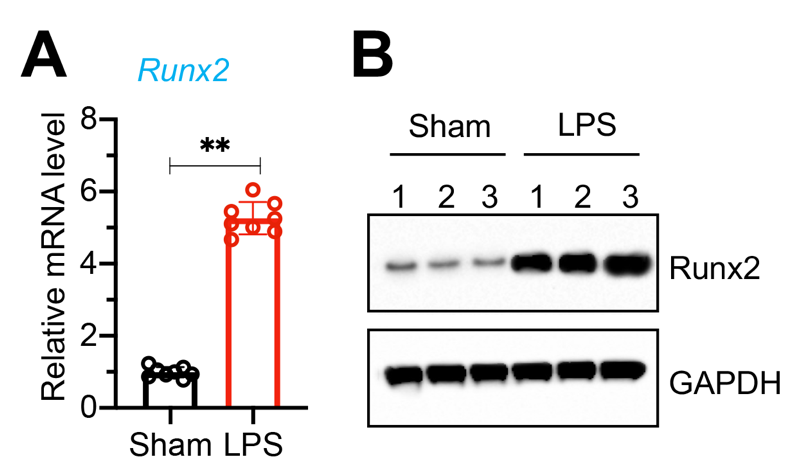
**

**Figure S11. Runx2 mRNA and protein levels in sham and LPS groups of mice**

**(A)** Expression levels of *Runx2* mRNA in mice from sham and LPS-treated groups. RNA was isolated from IVDs of the same sham and LPS-challenged mice used in Figure S1, followed by RT-qPCR to quantify *Runx2* mRNA. **(B)** Expression levels of Runx2 protein in mice from sham and LPS-treated groups. Protein extracts from the same IVDs of sham and LPS-challenged mice used in Figure S1 were subjected to immunoblotting to measure the protein levels of Runx2. ***P* < 0.01.


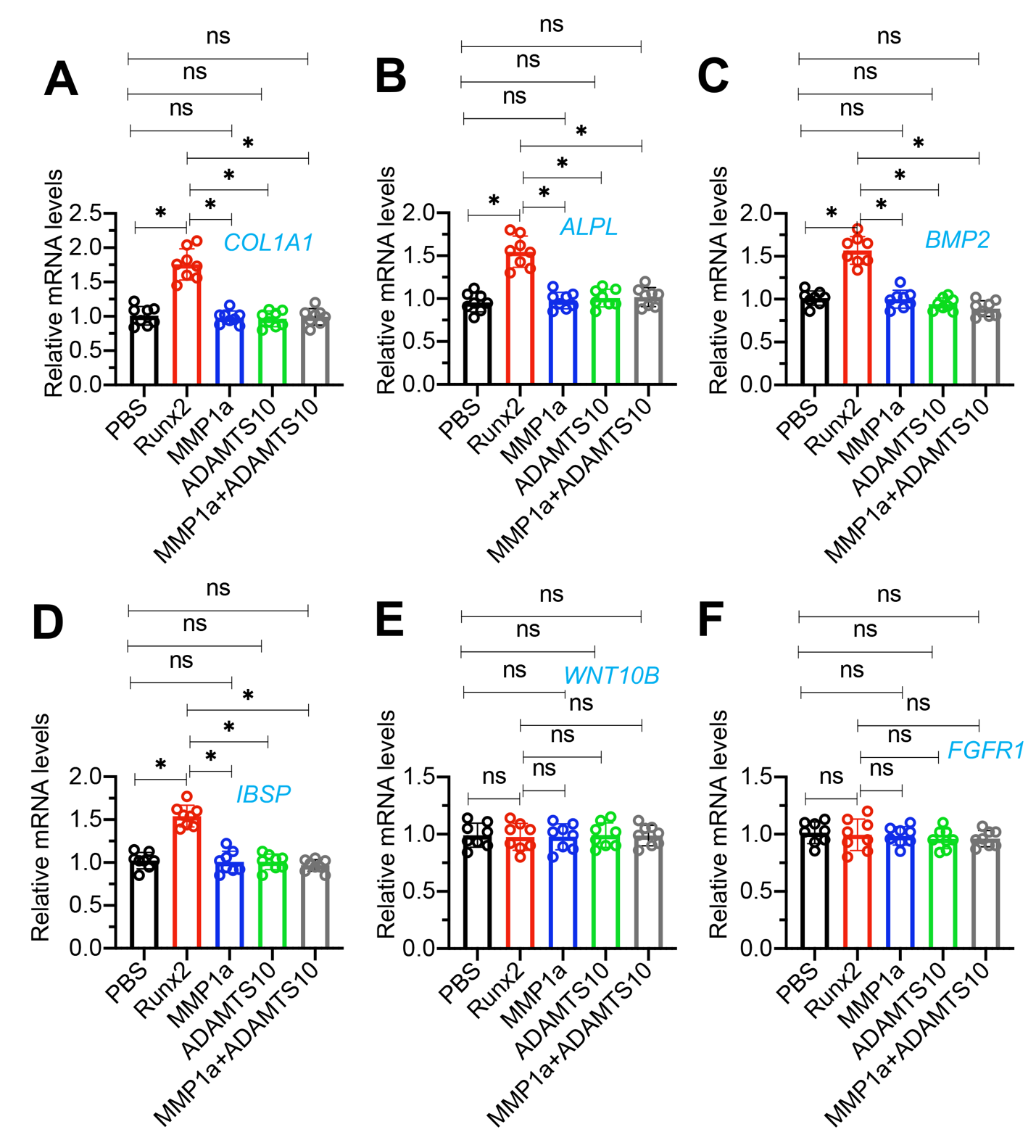


**Figure S12. Expression Levels of *COL1A1/ALPL/BMP2/IBSP/WNT10B/FGFR1* in mice administered PBS, recombinant Runx2, MMP1a, ADAMTS10, or MMP1a + ADAMTS10**

IVDs from mice treated with PBS, recombinant Runx2, MMP1a, ADAMTS10, or a combination of MMP1a and ADAMTS10 were collected for RNA isolation. RT-qPCR analyses were conducted to determine the mRNA expression levels of *COL1A1* **(A)**, *ALPL* **(B)**, *BMP2* **(C)**, *IBSP* **(D)**, *WNT10B* **(E)**, and *FGFR1* **(F)**. **P* < 0.05; ns represents no significant difference.


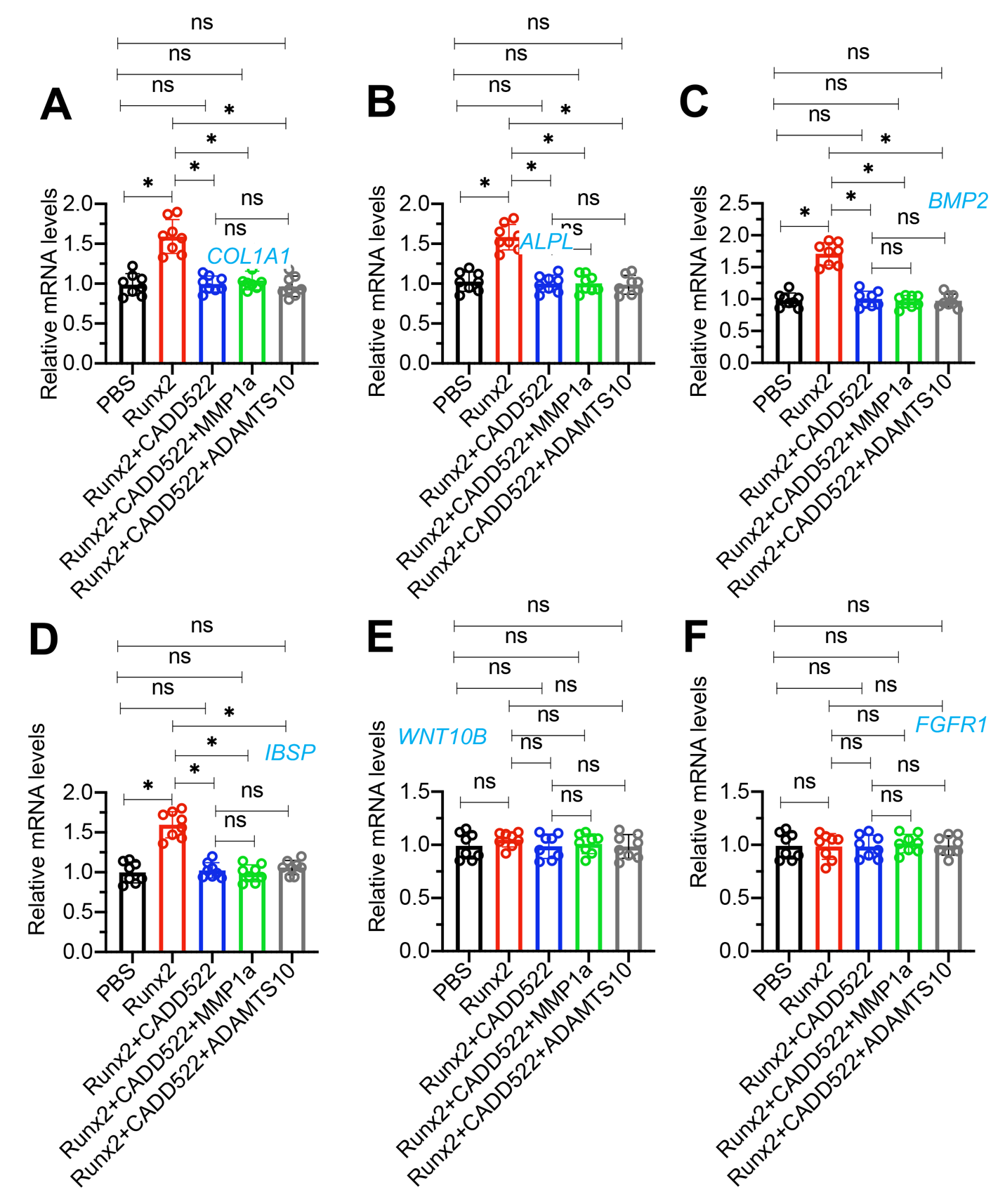


**Figure S13. Expression Levels of *COL1A1/ALPL/BMP2/IBSP/WNT10B/FGFR1* in mice administered PBS, recombinant Runx2, Runx2+CADD522, Runx2+CADD522+MMP1a, or Runx2+CADD522+ADAMTS10**

IVDs from mice treated with PBS, recombinant Runx2, Runx2+CADD522, Runx2+CADD522+MMP1a, or Runx2+CADD522+ADAMTS10 were collected for RNA isolation. RT-qPCR analyses were conducted to determine the mRNA expression levels of *COL1A1* **(A)**, *ALPL* **(B)**, *BMP2* **(C)**, *IBSP* **(D)**, *WNT10B* **(E)**, and *FGFR1* **(F)**. **P* < 0.05; ns represents no significant difference.


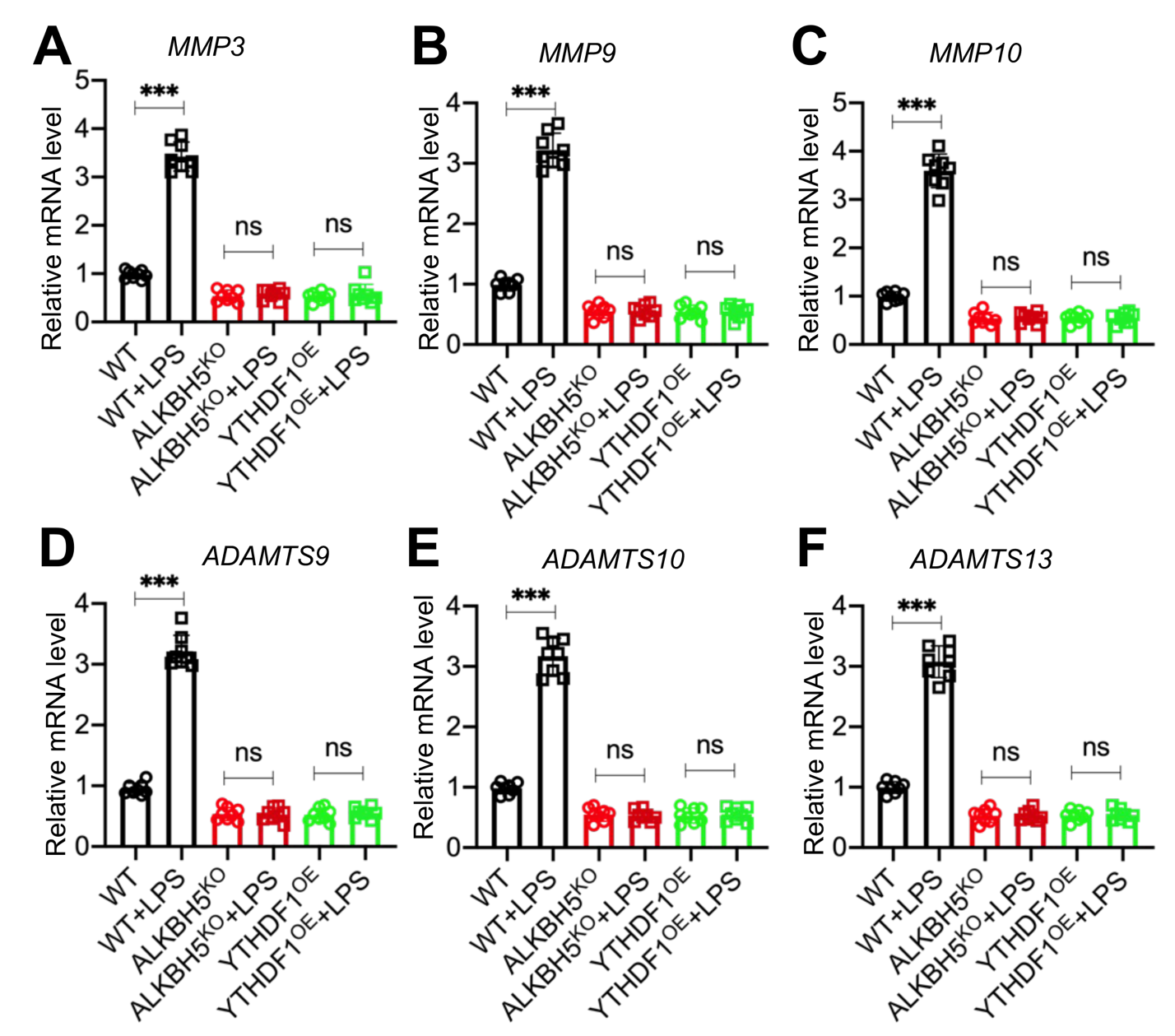


**Figure S14. Expression Levels of *MMP3/MMP9/MMP10/ADAMTS9/ADAMTS10/ADAMTS13* in LPS-treated ALKBH5^KO^ and YTHDF1^OE^ mice**

The same cDNA samples used in Figure 9I-9K were analyzed using RT-qPCR to quantify the mRNA expression levels of *MMP3* **(A)**, *MMP9* **(B)**, *MMP10* **(C)**, *ADAMTS9* **(D)**, *ADAMTS10* **(E)**, and *ADAMTS13* **(F)**. ****P* < 0.001; ns represents no significant difference.

**Table S1. Short hairpin RNAs (shRNAs) that specifically target genes**

| **Gene** | **TRC Clone ID** | **Sources** |
| --- | --- | --- |
| ALKBH5 | TRCN0000192524 | Sigma-Aldrich, China |
|  | TRCN0000201776 |  |
|  | TRCN0000039402 |  |
| Runx2 | TRCN0000095590 | Sigma-Aldrich, China |
|  | TRCN0000095592 |  |

**Table S2. Information of vectors for overexpression**

| **Vectors** | **Insertion sites** | **Forward primers (5’-3’)** | **Reverse primers (5’-3’)** |
| --- | --- | --- | --- |
| pCMV6-Entry-Flag | N/A | N/A | N/A |
| pCMV6-Entry-Flag-ALKBH5 | BamHI+NotI | CGGGATCCATGGCGGCCGCCAGCGGCTACA | ATTTGCGGCCGCTCAGTGTCTCCTCATCTTCACC |
| pCMV6-Entry-Flag-YTHDF1 | BamHI+NotI | CGGGATCCATGTCGGCCACCAGCGTGGACCC | ATTTGCGGCCGCTTATTGTTTGTTTCGATTCTGTCTT |

**Table S3. Primer pairs specifically targeting genes for RT-qPCR analyses**

| **Genes** | **Forward primers (5’-3’)** | **Reverse primers (5’-3’)** |
| --- | --- | --- |
| mALKBH5 | TGCTCCAGTGATCTGAGCCA | GTCCACTGCACAGTTCATACA |
| mMETTL3 | GACTACAGTGACCACCTTTG | GACCTTCTTGCTCTGCTGTTC |
| mMETTL14 | GCAGCTCCTAGCTCAGCAGT | TGGAGCAGATGTATCATAGGA |
| mWTAP | GCTTCAGCTCCAAGTACCAGC | ATCCAGACCCAGACGTCCTCT |
| mFTO | GCACCGATTGGTGGTGTGAG | GGATGGCAGACAGAATCTCA |
| mYTHDC1 | ATGCATTCTCAGCCTCGATC | TGGTCTCTGGTGAAACTCAG |
| mYTHDC2 | ACGAGGTGGTGGTGACATTC | GTTGACTGAGAACTGAAGTG |
| mYTHDF1 | TACTCCATCTGGTGTAGTACTG | TGGTGCCGTAGTCCACAG |
| mYTHDF2 | GACACTTCTGTGGAGTTGCAG | GTGTCGCAGTTGGCTATTGG |
| mYTHDF3 | CCAGTTACATGTACACTGAG | GTCTGATTAGACTGAGGTAC |
| mIGF2BP1 | AGCAGGTTGAGGCTGAGCT | TAACCATTCAGAGCCCAGAA |
| mIGF2BP2 | CAGCAGGTTGCAGACAGTGA | TGCCGGTGCTGTTCATTGAC |
| mIGF2BP3 | TCAGCGTGCAGAGCCAGTAGA | TCACTCAGTTCACCACACTCA |
| mMMP1a | GCTAGTATGATAGATCAAGTC | CCTCACAAACAGCAGCATCA |
| mMMP2 | TGTGCGACCACAACCAACTAC | TTGGTGTAGGTGTAGATCGG |
| mMMP3 | CAGTCTTGGCTCATGCCTATG | CTTGGCTGAGTGGTAGAGT |
| mMMP9 | ACTCACACGACATCTTCCAG | AGGAGGTCGTAGGTCACGTA |
| mMMP10 | AGTCTACAGGTTCTCCACAA | CAGGTCTTGGAGAGACAGA |
| mMMP12 | AGGTATGATGTGAGGCAGGAG | GTCATATTCCAATTGATAGGCT |
| mMMP15 | CAACTTCGACACAGTGGCTGT | TCGTAGGCAGCACTGATGTTC |
| mADAMTS9 | CTGCAGAGCTCAGGACAAATG | TTGCTGTCATCGTGAGGCAT |
| mADAMTS10 | AGCTCATGGCTGCCCACATT | TTGGGTACACGAAGTCCT |
| mADAMTS13 | ACACATTGTCAACCGCAGGC | TGTGAATCTCCTCTAAGTG |
| mADAMTS14 | TATGCAGGAACACTTCCAG | TGGACCTCCGCCTTCACTGT |
| mADAMTS20 | TCAAGGACATGCGGAGGTGG | CCGTCGAAGTCGGAGCATT |
| mβ-Actin | CATTGCTGACAGGATGCAGAAGG | TGCTGGAAGGTGGACAGTGAGG |
| hALKBH5 | CATACGGCCTCAGGACATCAA | TCAGAGCAGGGTCCCTGTTG |
| hRunx2 | AGCAGTGGCCCAGTGGTAT | ATCATGACTGAGTGAGTG |
| hMMP1 | CAGCTAGCTCAGGATGACA | CCCGAATCGTAGTTATAG |
| hMMP2 | TGACGATGAGCTATGGACCT | TGTAGGTGGTGGAGCACCAGA |
| hMMP3 | AGCCAACTGTGATCCTGCT | GCCTGAAGGAAGAGATGGC |
| hMMP9 | ACGCAGACATCGTCATCCAGT | GACCACAACTCGTCATCGT |
| hMMP10 | TGACGTTGGTCACTTCAGCT | TTCCCAGACTTTCAGAGCT |
| hMMP12 | TGAGGATGTTGACTACGCA | ATGGGCTAGGATTCCACCT |
| hMMP15 | AGGACGTTGACAACTTCAAG | TTCCCACCTGGGGGTGGT |
| hADAMTS9 | GCCAGTGGCAGCATTCGAAG | CCACTATCTTCACTAATAG |
| hADAMTS10 | AGGACCCAGCCAAGCTCAT | GTACACAAAGTCCTGTCT |
| hADAMTS13 | ACCTTGAGCCAACAGGAAC | CCCCAAAGCAGCAACATGT |
| hADAMTS14 | CAGACCTGCTTGGCATTCAG | GATGCAGTGACCTTTGAAG |
| hADAMTS20 | CTCTATTGTCAGGTTGCTG | ACACGTGATCACAACCAGCT |
| hYTHDF1 | TCACAGCGACACCCTCAGCA | CAGAAAGGACACCAGTCAGT |
| hβ-Actin | TGAAGTGTGACGTGGACAT | TCTTGATCTTCATTGTGCT |

**Table S4. Antibodies used for western blotting**

| **Antibodies** | **Catalog numbers** | **Dilution fold** | **Sources** |
| --- | --- | --- | --- |
| Anti-ALKBH5 | #703570 | 1:2500 | Thermo Fisher |
| Anti-METTL3 | #MA5-27527 | 1:3000 | Thermo Fisher |
| Anti-METTL14 | #PA5-117138 | 1:3000 | Thermo Fisher |
| Anti-WTAP | #60188-1-IG | 1:2000 | Thermo Fisher |
| Anti-FTO | #PA5-89791 | 1:3000 | Thermo Fisher |
| Anti-YTHDC1 | #PA5-76212 | 1:2000 | Thermo Fisher |
| Anti-YTHDC2 | #MA5-47205 | 1:2500 | Thermo Fisher |
| Anti-YTHDF1 | #ab220162 | 1:2000 | Abcam, Shanghai, China |
| Anti-YTHDF2 | #ab220163 | 1:3000 | Abcam |
| Anti-YTHDF3 | #ab220161 | 1:3500 | Abcam |
| Anti-IGF2BP1 | #703103 | 1:2500 | Thermo Fisher |
| Anti-IGF2BP2 | #11601-1-AP | 1:3000 | Thermo Fisher |
| Anti-IGF2BP3 | #MA5-27480 | 1:2500 | Thermo Fisher |
| Anti-Runx2 | #sc-390715 | 1:2500 | Santa Cruz Biotechnology, Shanghai, China |
| Anti-MMP1 | #ab137332 | 1:1500 | Abcam |
| Anti-MMP2 | #436000 | 1:2000 | Thermo Fisher |
| Anti-MMP3 | #MA5-17123 | 1:1500 | Thermo Fisher |
| Anti-MMP9 | #10375-2-AP | 1:2500 | Thermo Fisher |
| Anti-MMP10 | #ab261733 | 1:2000 | Abcam |
| Anti-MMP12 | #sc-390863 | 1:1500 | Santa Cruz Biotechnology |
| Anti-MMP15 | #PA5-75175 | 1:1500 | Thermo Fisher |
| Anti-ADAMTS9 | #PA1-1760 | 1:2000 | Thermo Fisher |
| Anti-ADAMTS10 | #PA5-103575 | 1:2500 | Thermo Fisher |
| Anti-ADAMTS13 | #MA5-42805 | 1:2000 | Thermo Fisher |
| Anti-ADAMTS14 | #PA5-48070 | 1:1500 | Thermo Fisher |
| Anti-ADAMTS20 | #PA5-103582 | 1:2000 | Thermo Fisher |
| goat anti-mouse IgG H&L | #ab6789 | 1:8000 | Abcam |
| HRP anti-Rabbit IgG antibody | #ab288151 | 1:8000 | Abcam |

**Table S5. Primers used for MeRIP RT-qPCR**

| **Gene** | **Forward primers (5’-3’)** | **Reverse primers (5’-3’)** |
| --- | --- | --- |
| Runx2 | ACCGTCAAAGGTGTTGTCTG | CACACAGCAGAGGCTGTGGTTT |

**Table S6. Primers used for ChIP RT-qPCR analyses**

| **Gene promoters** | **Forward primers (5’-3’)** | **Reverse primers (5’-3’)** |
| --- | --- | --- |
| MMP1a | GTGAGATCCTCTCTCAGTTT | GGATGTCTGCCCTCATGGAG |
| MMP2 | CAGATCACATTCCTCTCG | CATGTCTCCTACTACGT |
| MMP3 | ATCGACAGCATAGTGTGCT | CCAGTAGAGTCTCACT |
| MMP9 | TCCCACAAAGTCTGCAGT | TGTGTGTGTTTACACTACC |
| MMP12 | CTATGCACTAGGCTCTTC | TGGAATGAGGCTGCTGTGT |
| ADAMTS9 | CTTACCAAGGAGCTGTCT | CCTAGCCCTTCTGAAAGT |
| ADAMTS10 | ACGTAGAGACAGAGAACTA | GGGGTAATGACAGCCT |
| ADAMTS13 | TGCTGGAGACACTTAAC | CCTGATGAGAGACTTGTT |

**Table S7. The basic information of all human participants in this study**

| **Participants** | **Average ages** | **Gender** | **Degenerative IVDs** |
| --- | --- | --- | --- |
| Control | 30.52 ±5.36 | 4F/6M | No |
| IDD | 63.76 ±10.21 | 5M/5F | Yes |

**Table S8. Differentially expressed genes in ALKBH5-KD cells**

| **Genes** | **Control-KD1** | **Control-KD2** | **ALKBH5-KD1** | **ALKBH5-KD2** |
| --- | --- | --- | --- | --- |
| MAML1 | -13.2 | -9.1 | 9.3 | 7.5 |
| MCM6 | -11.8 | -7.6 | 8.3 | 9.8 |
| IFIH1 | -10.9 | -10.3 | 7.4 | 7.1 |
| IBSP | -10.2 | -6.4 | 6.7 | 6.5 |
| CLCN5 | -9.1 | -9.2 | 8.4 | 8.7 |
| P3H1 | -8.5 | -8.7 | 9.1 | 7.2 |
| VDR | -7.9 | -5.4 | 8.8 | 6.5 |
| ACP1 | -7.2 | -4.3 | 7.3 | 7.9 |
| FBN1 | -7 | -7.8 | 5.4 | 4.6 |
| PPIB | -6.7 | -8.3 | 7.8 | 4.2 |
| PLS3 | -6.2 | -6.5 | 3.6 | 6.7 |
| MEG3 | -6 | -7.9 | 7.3 | 8.2 |
| FLT3 | -5.5 | -5.2 | 5.5 | 4.3 |
| EXT1 | -5.3 | -7.3 | 6.4 | 6.5 |
| SRP72 | -4.5 | -8.2 | 6.3 | 3.7 |
| MSX2 | -4 | -5.7 | 8.6 | 6.8 |
| FN1 | -3.2 | -7.1 | 7.6 | 3.2 |
| EXT2 | -3 | -5.5 | 4.6 | 4.6 |
| Runx2 | 10.4 | 8.3 | -11.1 | -12.3 |
| CCN1 | 10.1 | 9.2 | -7.3 | -5.6 |
| PSME2 | 9.3 | 5.6 | -6.5 | -9.2 |
| MMP1 | 9.1 | 7.8 | -8.2 | -7.6 |
| MMP2 | 8.5 | 5.2 | -4.5 | -8.4 |
| MMP3 | 8.2 | 3.6 | -7.6 | -9.2 |
| MMP7 | 7.9 | 5.7 | -8.2 | -5.5 |
| MMP9 | 7.5 | 8.3 | -6.5 | -7.6 |
| MMP10 | 7.1 | 9.2 | -7.7 | -8.2 |
| MMP13 | 6.7 | 10.3 | -9.2 | -9.2 |
| MMP17 | 6.4 | 5.7 | -6.8 | -8.4 |
| ADAMTS1 | 6 | 8.3 | -7.2 | -5.7 |
| ADAMTS5 | 5.6 | 6.3 | -5.4 | -4.6 |
| ADAMTS8 | 5.3 | 5.1 | -6.2 | -7.3 |
| ADAMTS14 | 5.1 | 4.8 | -7.6 | -8.2 |
| ADAMTS15 | 4.8 | 6.7 | -5.4 | -5.1 |
| ITGB1 | 4.6 | 8.2 | -7.6 | -4.5 |
| SKP1 | 4.3 | 7.2 | -5.1 | -7.8 |
| CDK2 | 4 | 5.4 | -8.6 | -3.5 |
| DLX6 | 3.5 | 6.5 | -9.2 | -4.1 |
| BRD2 | 3.4 | 7.2 | -5.5 | -3.7 |
