# Supplementary material for: ALKBH5-mediated m6A demethylation of Runx2 mRNA promotes extracellular matrix degradation and intervertebral disc degeneration
Source: Cell Biosci. 2024 Jun 14;14:79. doi: 10.1186/s13578-024-01264-y (PMC11179301; doi:10.1186/s13578-024-01264-y)

Figure 10T

Anti-ALKBH5


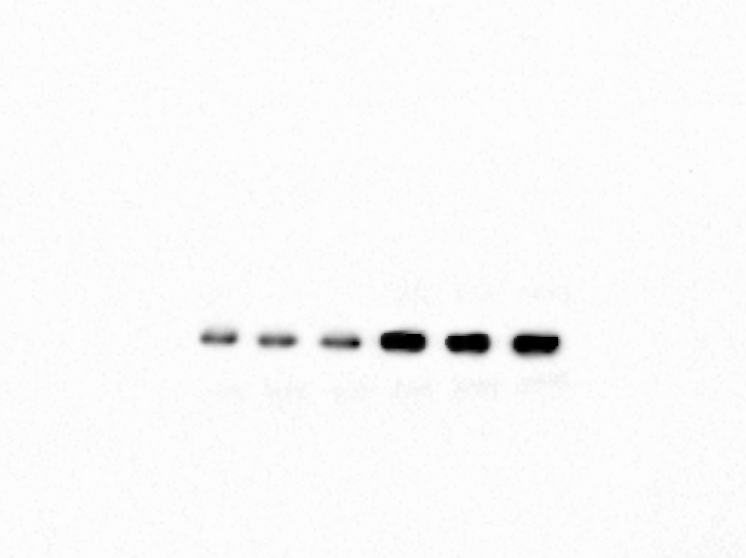


Anti-Runx2


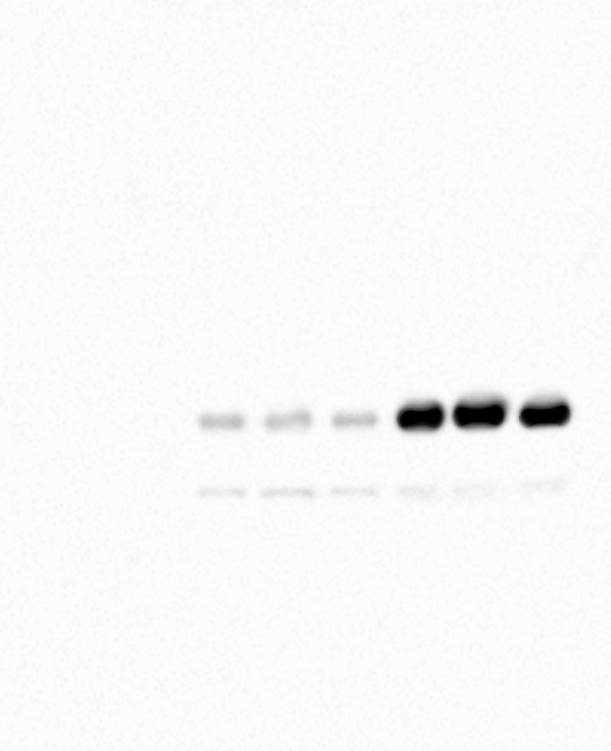


Anti-MMP1


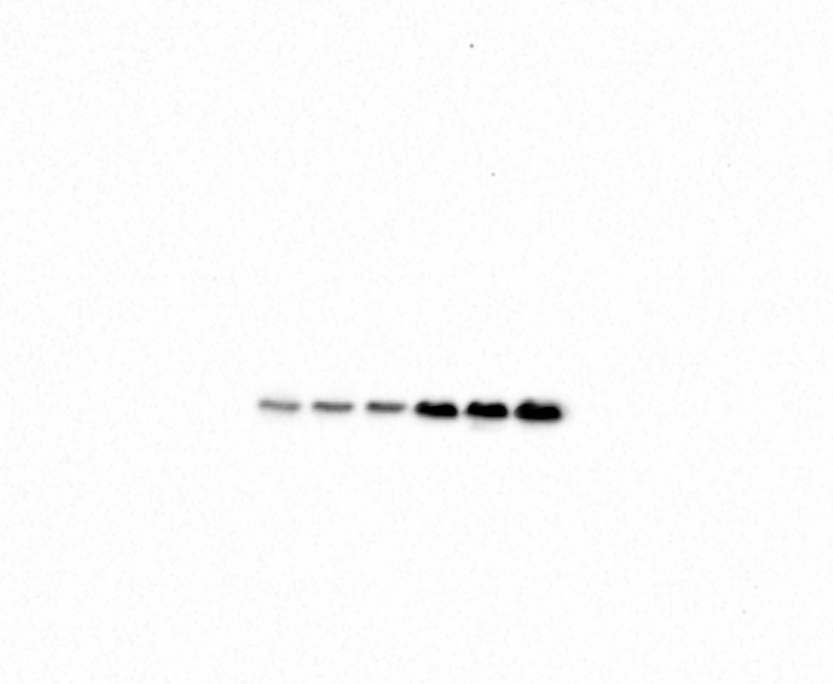


Anti-MMP2


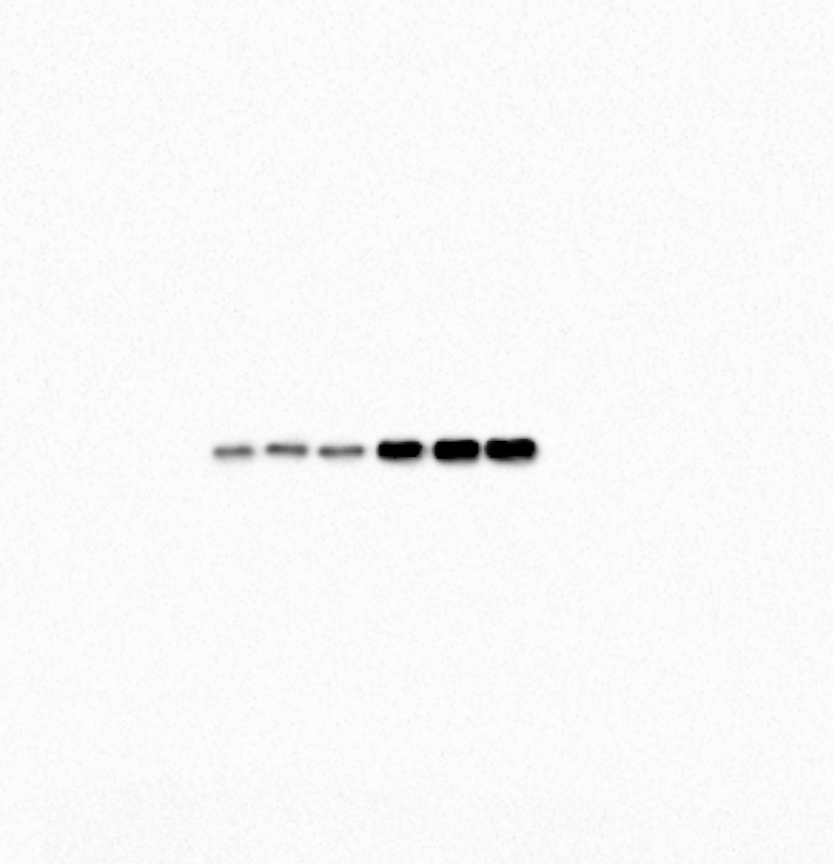


Anti-MMP3


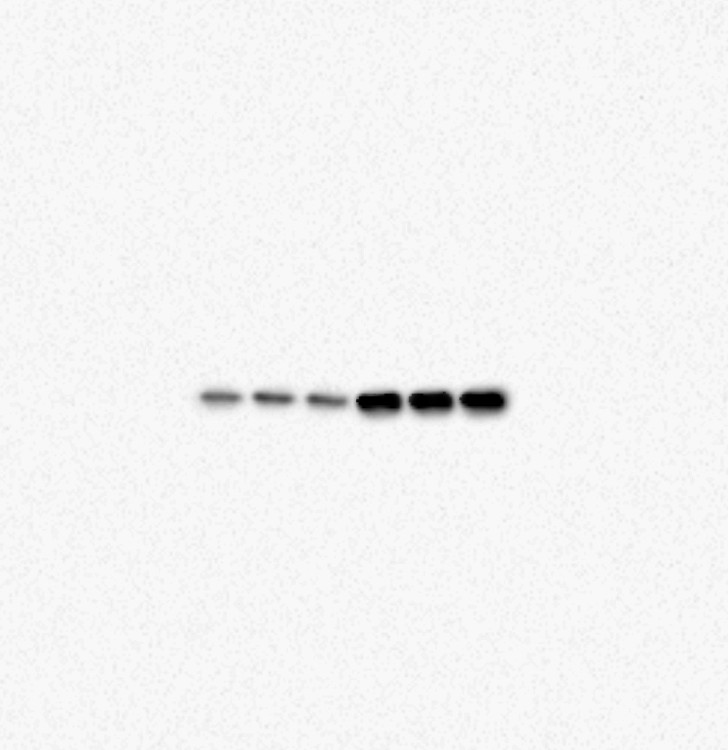


Anti-MMP9


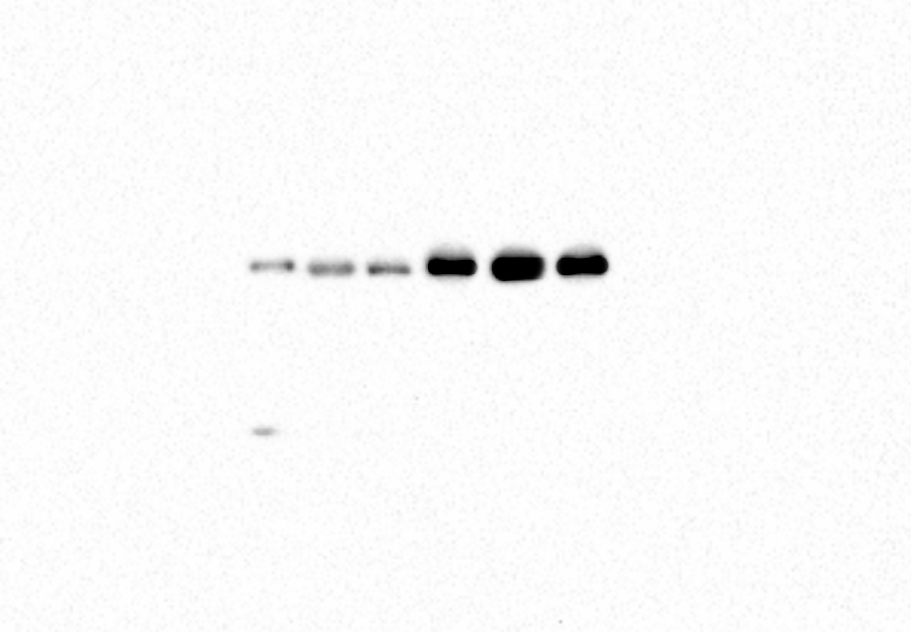


Anti-MMP10


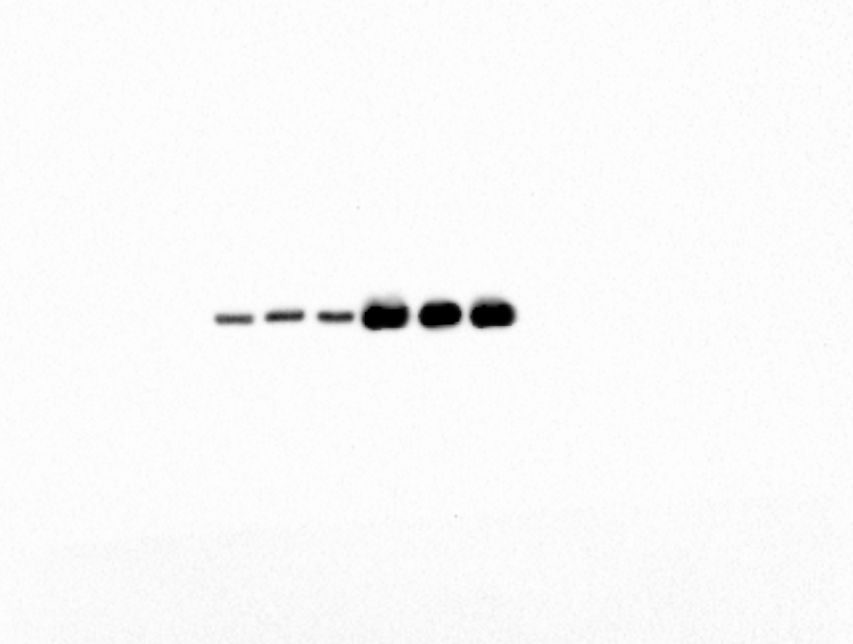


Anti-MMP12


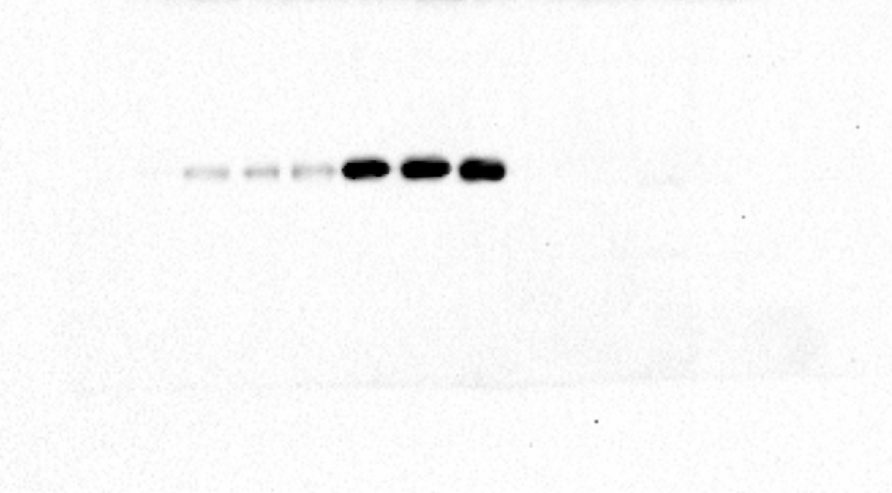


Anti-MMP15


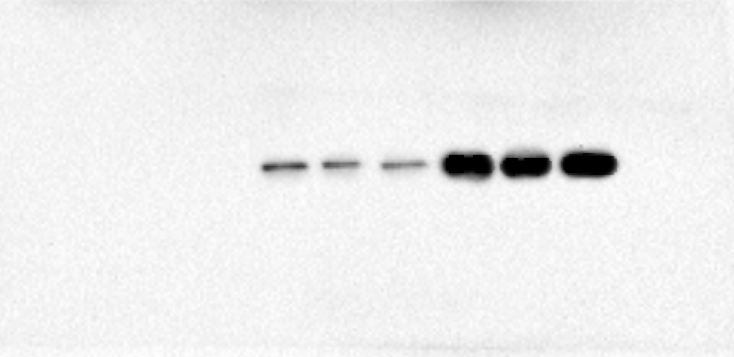


Anti-ADAMTS9


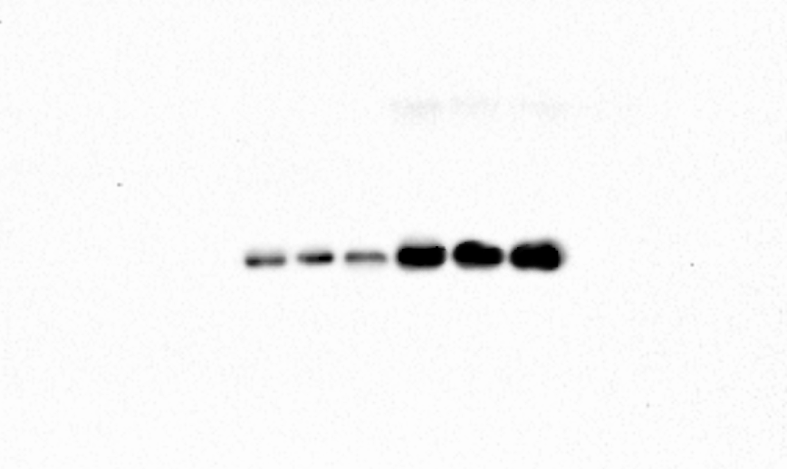


Anti-ADAMTS10


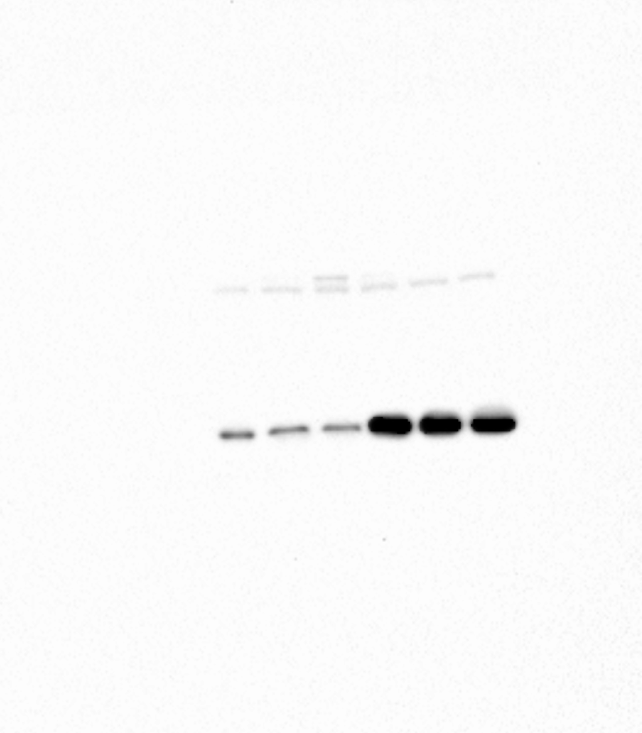


Anti-ADAMTS13


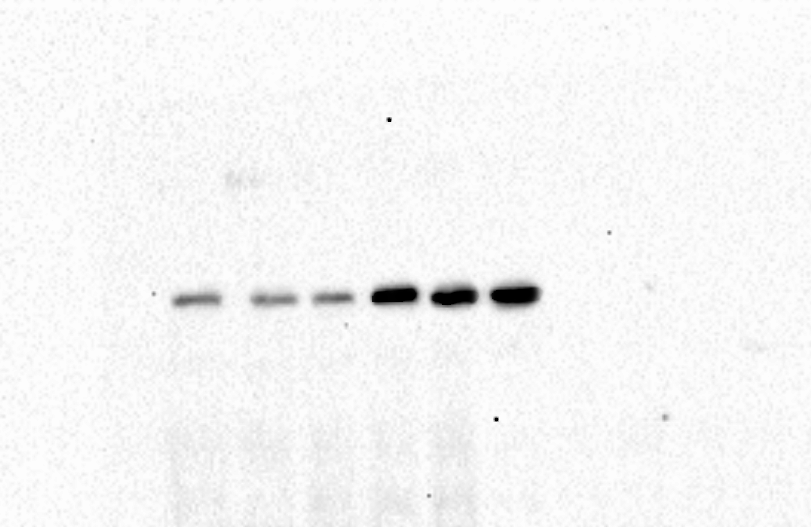


Anti-ADAMTS14


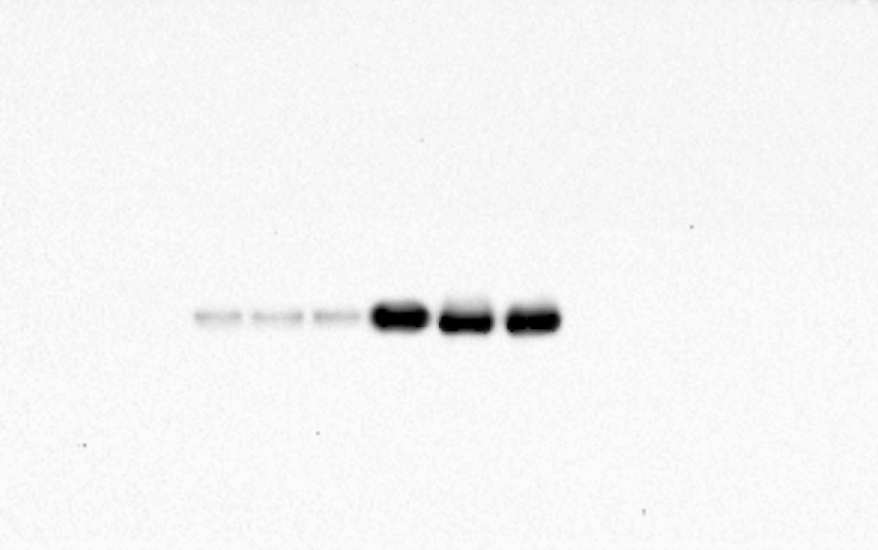


Anti-ADAMTS20


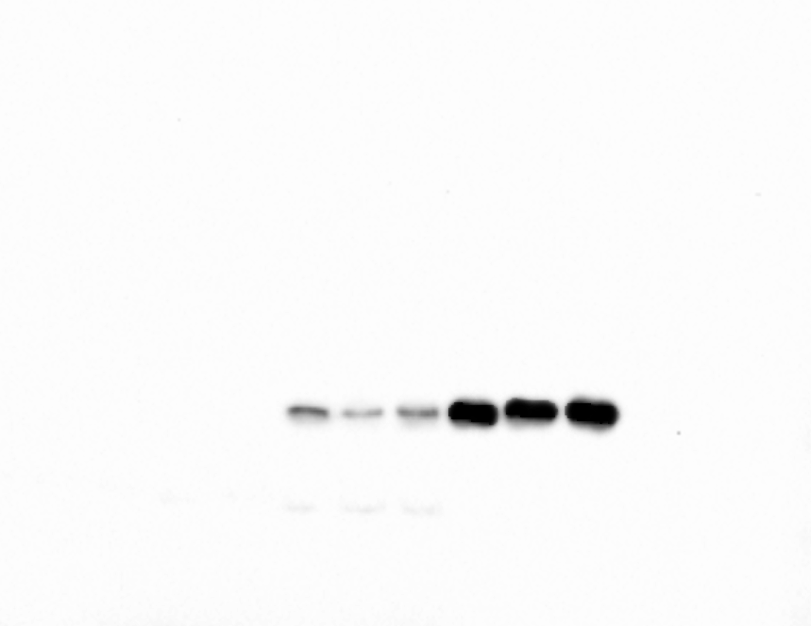


Anti-YTHDF1


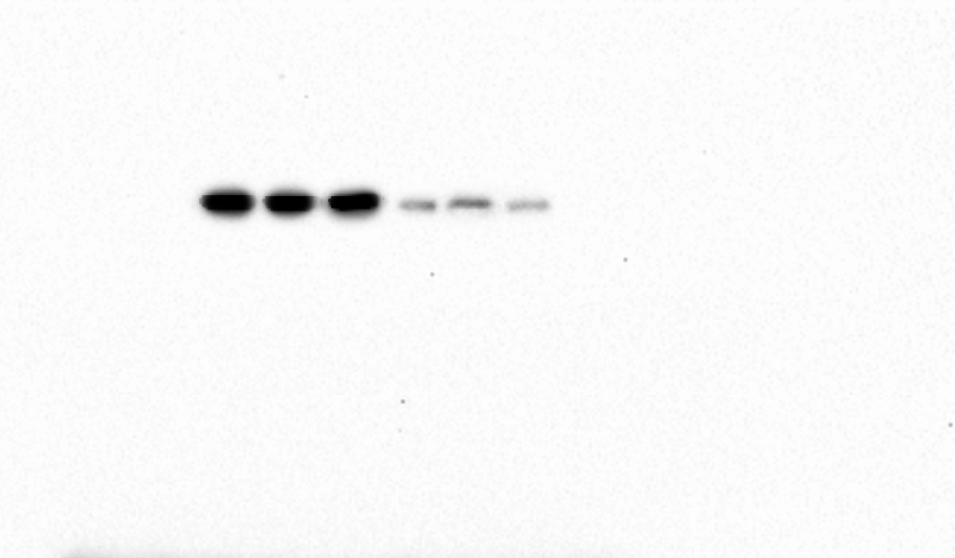


Anti-GAPDH


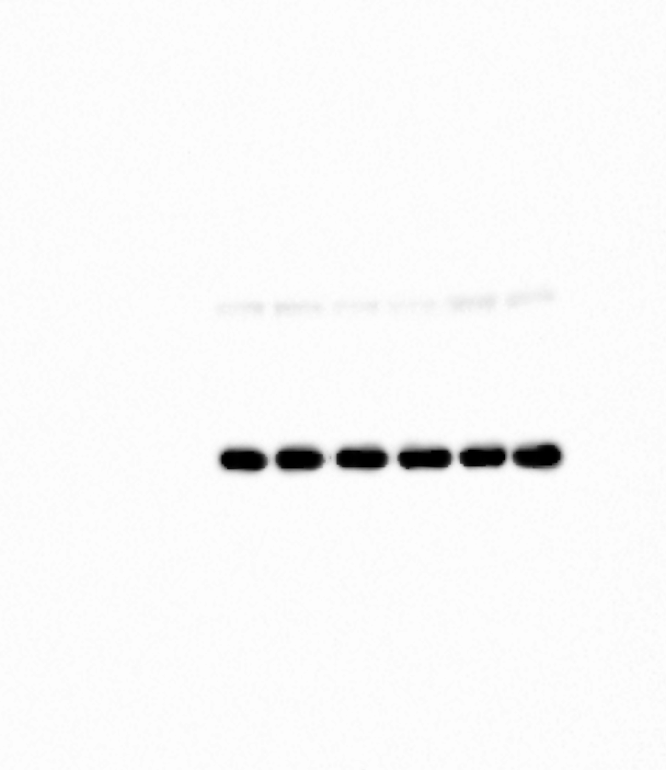

Supplement: Supplementary file 2 — Supplementary Material 2 [file 13578_2024_1264_MOESM2_ESM.docx]
